# Colloidal Synthesis of (PbBr_2_)_2_(AMTP)_2_PbBr_4_ a Periodic Perovskite “Heterostructured” Nanocrystal

**DOI:** 10.1021/acs.cgd.3c01472

**Published:** 2024-03-26

**Authors:** Emma H. Massasa, Lotte T. J. Kortstee, Rachel Lifer, Saar Shaek, Boaz Pokroy, Ivano E. Castelli, Yehonadav Bekenstein

**Affiliations:** † Department of Materials Science and Engineering, Technion— Israel Institute of Technology, 32000 Haifa, Israel; ‡ The Solid-State Institute, Technion—Israel Institute of Technology, 32000 Haifa, Israel; § Department of Energy Conversion and Storage (DTU Energy), Technical University of Denmark, Anker Engelunds Vej 411, DK-2800 Kongens Lyngby, Denmark

## Abstract

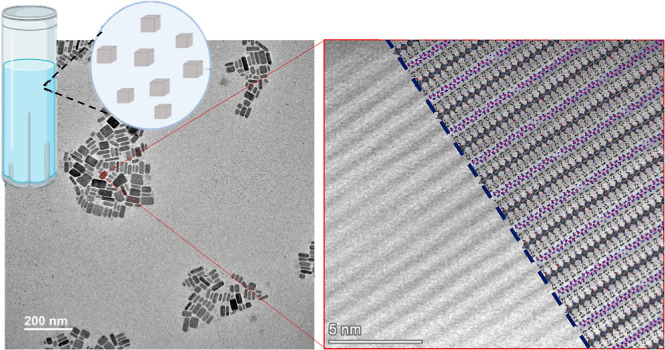

Heterostructures in nanoparticles challenge our common understanding of interfaces due to quantum confinement and size effects, giving rise to synergistic properties. An alternating heterostructure in which multiple and reoccurring interfaces appear in a single nanocrystal is hypothesized to accentuate such properties. We present a colloidal synthesis for perovskite layered heterostructure nanoparticles with a (PbBr_2_)_2_(AMTP)_2_PbBr_4_ composition. By varying the synthetic parameters, such as synthesis temperature, solvent, and selection of precursors, we control particle size, shape, and product priority. The structures are validated by X-ray and electron diffraction techniques. The heterostructure nanoparticles’ main optical feature is a broad emission peak, showing the same range of wavelengths compared to the bulk sample.

## Abstract 2

We have developed a colloidal synthesis for (PbBr_2_)_2_(AMTP)_2_PbBr_4_ heterostructure nanocrystals, demonstrating the ability to control the shape and size of the particles by adjusting synthesis parameters and analyzing their optical properties, with confirmation of the heterostructure achieved through X-ray and electron diffraction techniques.

## 1 Introduction

A heterostructure is defined as an interface between two different materials. Heterostructure interfaces are interesting since they introduce synergistic properties, sometimes lacking in the parent materials.^1−9^ Perovskite heterostructures have the potential to influence optoelectronic devices deeply; they can influence the electronic structure via band alignment^10,11^ or affect device functionality through charge transfer or selectivity.^12,13^ Another important contribution of the perovskite heterostructure is stability, which can be improved through surface passivation and chemical reactivity.^14,15^ 3D perovskites can degrade in humidity and heat due to the high polarity of the A cation (in organic perovskites) and relatively easy ion migration. Structural barriers for ion migration, such as those in heterointerfaces, can reduce the degradation process and stabilize the structure.^16^ However, for perovskite nanocrystals, uniform growth of interfaces is challenging due to low melting temperatures, ionic bonds, and dynamic ligands on the surface of the nanoparticles. The perovskite nanoparticles tend to degrade before forming the interface. There have been reports of lead halide perovskite interfaces with Pb chalcogenides,^17−21^ ZnS,^15^ Cs_4_PbBr_6_,^22^ and between perovskite/metal oxides^23^ and perovskite/metals.^24^ Other examples of perovskite heterostructures are Ruddlesden–Popper lead halide perovskites nanosheets,^25^ two-dimensional halide organic–inorganic perovskite lateral epitaxial heterostrucutures,^26^ and thin films of halide perovskite and oxide perovskite heterostructures.^27^ However, as nanoparticles, aside from a few examples, this results in an uncontrolled growth of small decorations or islands rather than uniform growth.

**Figure 1:**
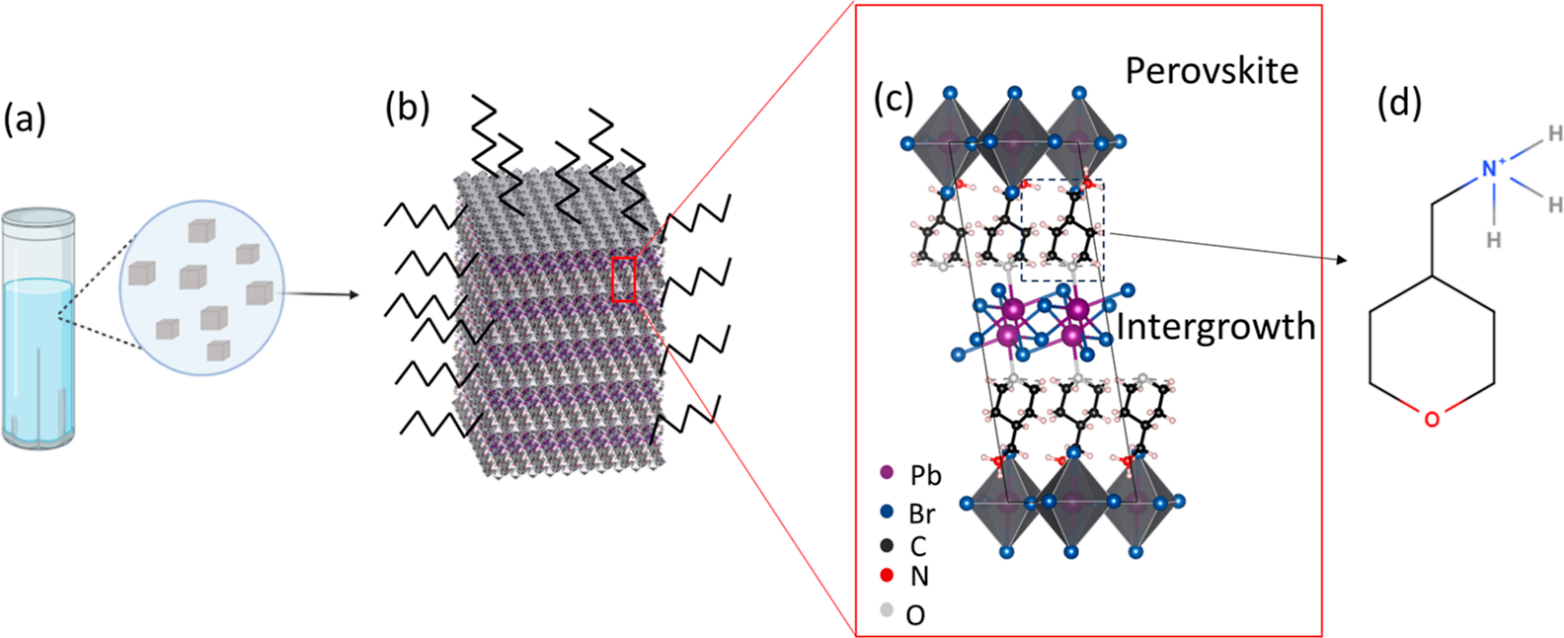
(a) Schematic of a colloidal solution. (b) Schematic of the colloidal heterostructure nanoparticle with (PbBr_2_)_2_(AMTP)_2_PbBr_4_ composition and oleic acid/oleylamine surface ligands. (c) Unit cell of the (PbBr_2_)_2_(AMTP)_2_PbBr_4_ heterostructure composed of perovskite and intergrowth layers. (d) Schematics of 4-Aminomethyltetrahydropyran (AMTP) molecule.

Recent advancement in the field was made by Aubrey et al. by creating a heterostructure with a (PbBr_2_)_2_(AMTP)_2_PbBr_4_ composition, where an interface between a perovskite layer and an intergrowth layer is placed as part of the material itself.^28^ This heterostructure is built from two repeating layers, a PbBr_4_ perovskite layer and an intergrowth layer of (PbBr_2_)_2_(AMTP)_2_, as seen in Figure 1. The intergrowth layer comprises an organic linker of 4-(ammoniomethyl)-tetrahydropyran (AMTP) with an ammonium tail acting as the A site cation of the perovskite structure, leading to electrostatic bonds between the two layers. In the bulk form, the periodic heterostructure was shown to support anisotropic emission and photocurrent due to the layered strcuture.^9,29^

In this work, we developed a colloidal synthesis that supports the nucleation of periodic heterostructures and their growth into nanocrystals with distinct sizes. We find a connection between the protonation of the amine precursors and the ability to form charge-neutral nuclei due to the structure of the alternating charge period layers.

## 2 Methods

### 2.1 Materials

Oleic acid (OA, 90%, Aldrich), PbBr_2_ (99.999%, Aldrich), oleyl amine (OAM, 98%, Aldrich), hexane (AR, bio laboratories), acetone (AR, bio laboratories), dimethyl sulfoxide (DMSO, 99.5%, Aldrich), octadecene (ODE, 90%, Aldrich), hydrobromic acid (HBr, 47%, AR), 4-aminomethyltetrahydropyran (AMTP, 97%, Acros Organics), and acetonitrile (anhydrous, 99.8%, Alfa Aesar) were used.

### 2.2 Synthesis

#### 2.2.1 Oleyl Ammonium Bromide (OAM-Br) Precursor

5 mL of acetonitrile is heated to 60 °C in a glass vial while stirring, and then 5 mL of OAM is added. When the solution becomes clear, 5.5 mL of HBr is slowly added dropwise. The solution is centrifuged at 3000 rpm for 5 min, the liquid is thrown away, and the precipitate is washed multiple times in acetonitrile via the centrifuge (using the same conditions) until a white wet powder is achieved. The powder is then put in a desiccator for at least 12 h to dry.

#### 2.2.2 Protonated AMTP Precursor

500 μL of HBr is mixed with 3 mL of acetonitrile at room temperature, and then 400 μL of AMTP is added. After the solution turns turbid, it is centrifuged at 3000 rpm for 5 min, the liquid is thrown away, and the precipitate is washed multiple times in acetonitrile via the centrifuge (using the same conditions) until a white wet powder is achieved. The powder is then put in a desiccator for at least 12 h to dry.

#### 2.2.3 Heterostructure Synthesis Using the Deprotonated Precursor

110 mg of PbBr_2_, 35 μL of AMTP, and 10 μL of HBr are mixed in a small glass vial with 0.5 mL of DMSO. In a separate glass vial, 5 mL of acetone, 120 mg of the OAM-Br precursor, and 0.5 mL of OA are mixed. After the regents are fully dissolved, the content of the first vial is injected into the second vial. The solution is stirred for 30 min, and then 5 mL of hexane is added, and the solution is taken straight to the centrifuge at 12,000 rpm for 5 min. The precipitate is redispersed in 5 mL of hexane.

#### 2.2.4 Heterostructure Synthesis Using the Deprotonated Precursor (in ODE)

110 mg portion of PbBr_2_, 35 μL of AMTP, and 10 μL of HBr are mixed in a small glass vial with 0.5 mL of DMSO at 120 °C until the solution turns blackish-yellow. In a separate glass vial, 5 mL of ODE, 180 mg of the OAM-Br precursor, and 0.5 mL of OA are mixed at 150 or 180 °C. After the regents are fully dissolved, the content of the first vial is injected into the second vial. The solution is stirred for 0–5 min, then the vial is taken off the heating plate, 5 mL of acetone is added, and the solution is taken straight to the centrifuge at 12,000 rpm for 5 min. The precipitate is redispersed in 5 mL of hexane.

#### 2.2.5 Heterostructure Synthesis Using the Protonated Precursor (in Acetone)

110 mg of PbBr_2_ (110 mg) and AMTP-Br (50 mg) are mixed in a small glass vial with 0.5 mL of DMSO. In a separate glass vial, 5 mL of acetone, 120 mg of the OAM-Br precursor, and 0.5 mL of OA are mixed (at room temperature or 50 °C). After the regents are fully dissolved, the content of the first vial is injected into the second vial. The solution is stirred for 30 min, and then 5 mL of hexane is added, and the solution is taken straight to the centrifuge at 12,000 rpm for 5 min, where the precipitate is redispersed in 5 mL of hexane.

#### 2.2.6 Heterostructure Synthesis Using the Protonated Precursor (in ODE)

110 mg of PbBr_2_ (110 mg) and AMTP-Br (50 mg) are mixed in a small glass vial with 0.5 mL of DMSO at room temperature or 120 °C until the solution turns orange. In a separate glass vial, 5 mL of ODE, 180 mg of the OAM-Br precursor, and 0.5 mL of OA are mixed at 120–150 °C. After the regents are fully dissolved, the content of the first vial is injected into the second vial. The solution is stirred for 0–15 min, the vial is taken off the hot plate to cool, 5 mL of acetone is added, and the solution is taken straight to the centrifuge at 12,000 rpm for 5 min. The precipitate is redispersed in 5 mL of hexane.

In the cleaned samples, the heterostructure products are cleaned with two centrifugations: first at 3500 rpm for 5 min and then at 7000 rpm for 10 min. In both, we kept the upper phase (the liquid).

#### 2.2.7 OAM_2_PbBr_4_ Monolayer Synthesis

110 mg portion of PbBr_2_ and 10 μL of HBr are mixed in a small glass vial with 0.5 mL of DMSO. In a separate glass vial, 5 mL of acetone, 0.5 mL of the OAM, and 0.5 mL of OA are mixed. After the reagents are fully dissolved, the content of the first vial is injected into the second vial. The solution is stirred for 30 min, then 5 mL of hexane is added, and the solution is taken straight to the centrifuge at 12,000 rpm for 5 min. The precipitate is redispersed in 5 mL of hexane.

#### 2.2.8 AMTP_2_PbBr_4_ Monolayer Synthesis

110 mg of PbBr_2_ (110 mg) and 35 μL of AMTP are mixed in a glass vial with acetone. The solution is stirred for 30 min, then 5 mL of hexane is added, and the solution is taken straight to the centrifuge at 12,000 rpm for 5 min. The precipitate is redispersed in 5 mL of hexane.

### 2.3 Characterization

#### 2.3.1 Transmission Electron Microscopy (TEM)

Samples for TEM imaging were prepared by drop-casting the colloidal solution on a Cu grid with a carbon film at room temperature. This research used a transmission electron microscope FEI Tecnai G2 T20 S-Twin TEM. The micrographs of the T-20 TEM were produced with a 200 kV acceleration voltage. In addition, we used a high-resolution transmission electron microscope FEI Titan 80–300 kV FEG-S/TEM. The micrographs were taken in the HRSTEM mode at a 200 keV acceleration voltage.

#### 2.3.2 X-ray Diffraction (XRD)

The samples were prepared by drop-casting the solution onto a glass slide. Measurements are taken using a Rigaku SmartLab 9 kW high-resolution diffractometer with 1.54 Å (Cu Kα) wavelength. The two-theta range of the measurements is 2θ = 1–60°, to include both sharp peaks on the monolayers in small angles and the heterostructure peak in small and wide angles. In-plane measurements were made on the same instrument, probing 2θχ while keeping φ constant.

In addition, high-resolution powder X-ray diffraction (XRD) measurements were carried out by the ID22 at a wavelength of 0.3542 Å at room temperature in the European Synchrotron Radiation Facility (ESRF), Grenoble, France. The samples were prepared by filling or smearing a borosilicate capillary with each sample. Rietveld refinement was carried out on the AMTP monolayer powder sample using GSAS-II software.

The XRD simulation was carried out using the software Crystal Maker, and the CIF was refined using the Rietveld refinement. The simulation was performed with a preferred orientation in the axis to achieve the diffractogram showing the stacked orientation of the monolayers.

#### 2.3.3 Optical Characterizations

Using a Biotek Synergy H1 plate reader, we measured the absorption of the heterostructure solutions. The solution was injected into a 96-well microplate, irradiated by using a xenon lamp (Xe900), and measured.

Additional measurements of photoluminescence (PL), excitation-photoluminescence emission (PLE), and PL–PLE maps were made using an Edinburgh FLS1000 PL spectrometer. Each sample solution is loaded into a quartz cuvette. The spectrometer is equipped with a xenon lamp light source suitable for low-emission intensity samples. The PL measurements were performed by exciting the sample at 350 nm.

Photoluminescence quantum yield (PLQY) was performed in an Edinburgh FLS1000 instrument using a cuvette and an integrated sphere holder.

Temperature-dependent PL was carried out on an Edinburgh FLS1000 PL spectrometer using a Nikon microscope with a temperature control Linkam stage. The samples were made via drop casting the colloidal solution onto a Si substrate.

#### 2.3.4 Density Functional Theory (DFT)

DFT calculations are performed using the Vienna Ab initio Simulation Package (VASP).^30,31^ Full lattice relaxations are performed at the generalized gradient approximation level, using the DFT-D3^32^ exchange–correlation function to correctly describe the van der Waals interactions between the organic components. The CIF file of the structure is obtained from the Rietveld refinement. A plane wave energy cutoff of 520 eV was set. A Γ-centered *k*-point mesh is automatically generated by VASP, using a 2 × 4 × 4 *k*-point mesh for both the hetero nano and bulk relaxations. Convergence is reached when the total energy difference between each consecutive ionic relaxation step is smaller than 5.0 × 10^–3^ eV. The electronic self-consistent loop is broken when the energy change between two electronic steps is smaller than 1.0 × 10^–6^ eV.

## 3 Results and Discussion

**Scheme 1:**
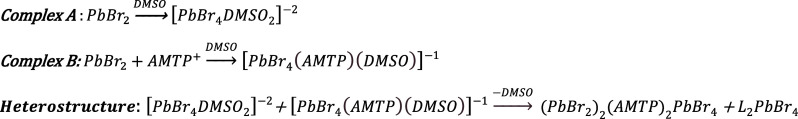
Hypothesized Chemical Reaction for the Heterostructure

The rationale of the synthesis is as follows: complexes A and B (Scheme 1) nucleate into the corresponding layer, perovskite, and intergrowth layers, respectively, resulting in the growth of the heterostructure. We hypothesize that the heterostructure will grow only when a charge-neutral nucleus is formed. Such nucleation is thermodynamically stable only when the two complexes are available to react. This is evident when the synthesis is held without protonation of the amine in the AMTP molecule. In this case, we see only the formation of perovskite monolayers. This suggests that the heterostructure nucleus could not form, probably due to the lack of charge neutrality the intergrowth layer provides the perovskite layer, or that the intergrowth layer does not form without the amine’s protonation. Therefore, to drive both complex reactions simultaneously, we synthesized a protonated precursor by protonating the amine in the AMTP molecule before the synthesis. An alternative synthetic path leaves the AMTP precursor deprotonated but introduces HBr into the reaction flask. Thus, according to our assumptions, we must create two different complexes in the solution to form two layers. The hypothesized chemical reaction is presented in Scheme 1.

We now explore the products of the two paths (the amine form, AMTP, and ammonium bromide form, AMTP^+^Br^–^, Figure S1a,b, respectively) that yield the heterostructure nanoparticles. In both routes, byproducts in the form of monolayers can be detected. See the Supporting Information for details.

**Figure 2:**
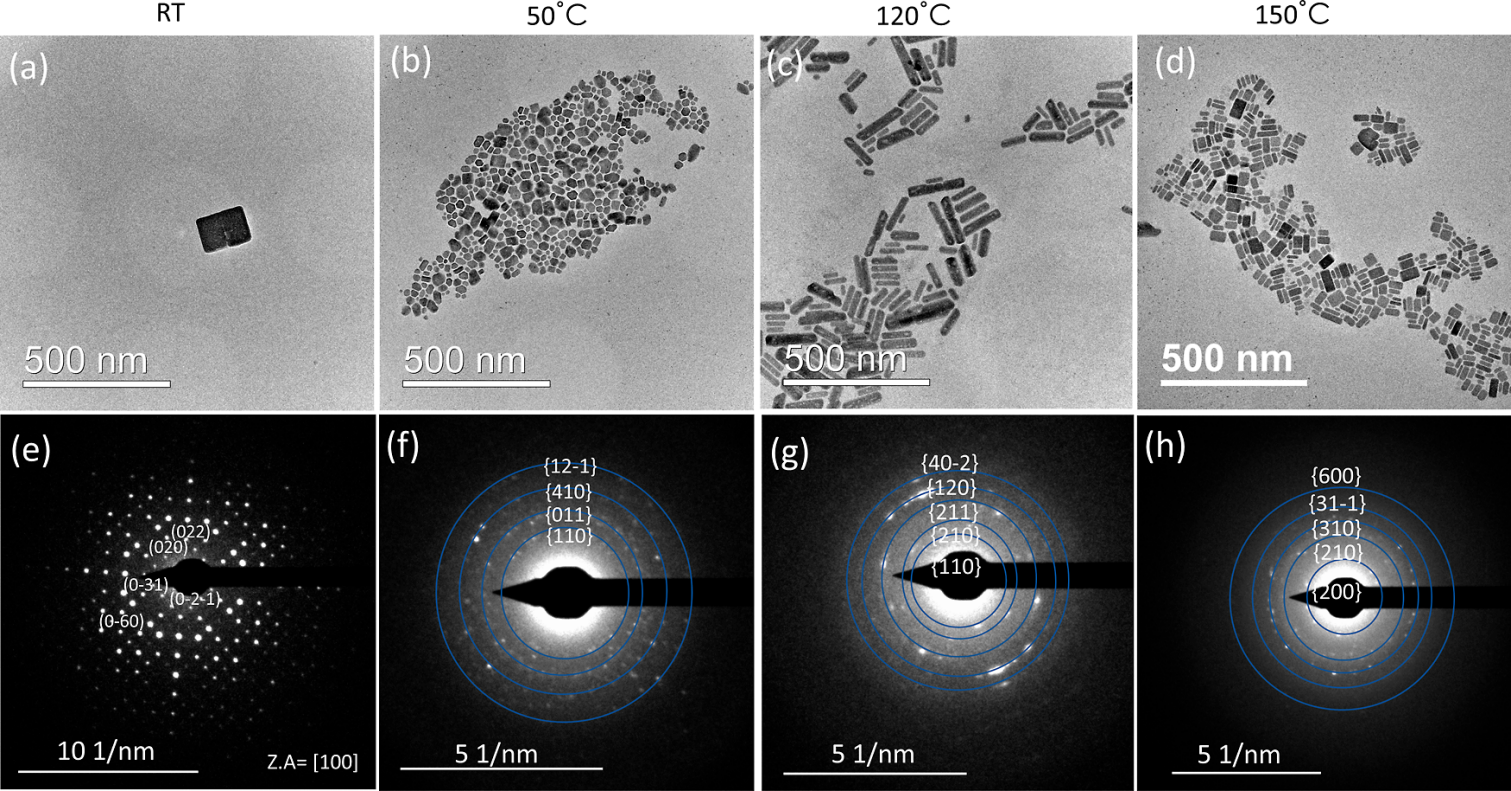
TEM micrographs and SAED of colloidal heterostructures using the protonated precursors with variations of reaction and growth temperature. (a) At room temperature, (b) 50 °C, both in acetone, (c) 120 °C, and (d) 150 °C, both in ODE. (e), (f), (g), and (h) are SAED of (a), (b), (c), and (d), respectively, matching the reported heterostructure diffractogram.

We explore the effects of the growth temperature on the synthesis products. Figure 2 presents colloidal heterostructures grown from the protonated precursor route with different synthesis parameters. The panels of the TEM micrographs (Figure 2a–d) present products grown at four different temperatures (and two different solvents, acetone and ODE). The nanocrystals’ corresponding selected area electron diffractions (SAED) confirm the exitance of the periodic heterostructure (Figure 2e–h).^28^ We show that by changing the temperature, we can control the average size, concentration, and uniformity of the nanoparticles, in agreement with classic nucleation theory. The theory predicts that a rise in temperature will reduce the critical radius of the nuclei, increasing the number of nuclei in the synthesis, overconsuming the monomer, and thus reducing the size of the nanoparticles. The change in particle size and uniformity of the nanoparticles with temperature is shown with size distributions in Figure S2. In this reaction path, the resulting nanoparticles are plates, as shown in Figure 2d.

**Figure 3:**
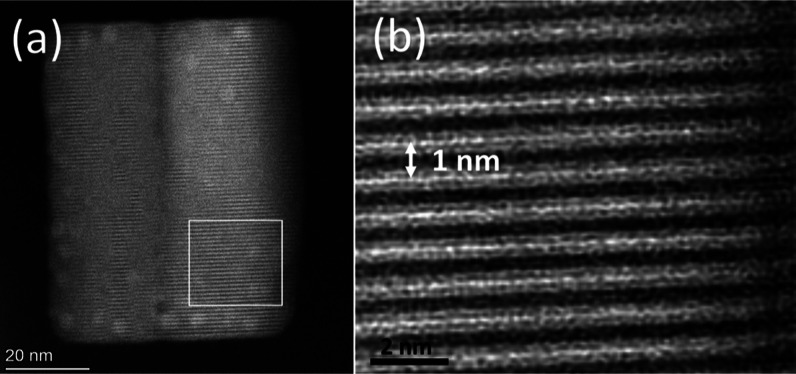
(a) Aberration-corrected HRSTEM micrograph of a heterostructure nanoparticle synthesized through the protonated route at 150 °C, and (b) zoom-in into the white square in (a), showing a *d* space matching the (200) plane.

Figure 3a presents an HRSTEM of a nanoparticle synthesized through the protonated route at 150 °C, and Figure 3b shows a zoom-in to the area marked with the white square showing crystal fringes with about 1 nm spacing matching the (200) *d*-space of the heterostructure that is equal to 1.09 nm.^28^ This further validates that the nanoparticles synthesized are the (PbBr_2_)_2_(AMTP)_2_PbBr_4_ heterostructure.

**Figure 4:**
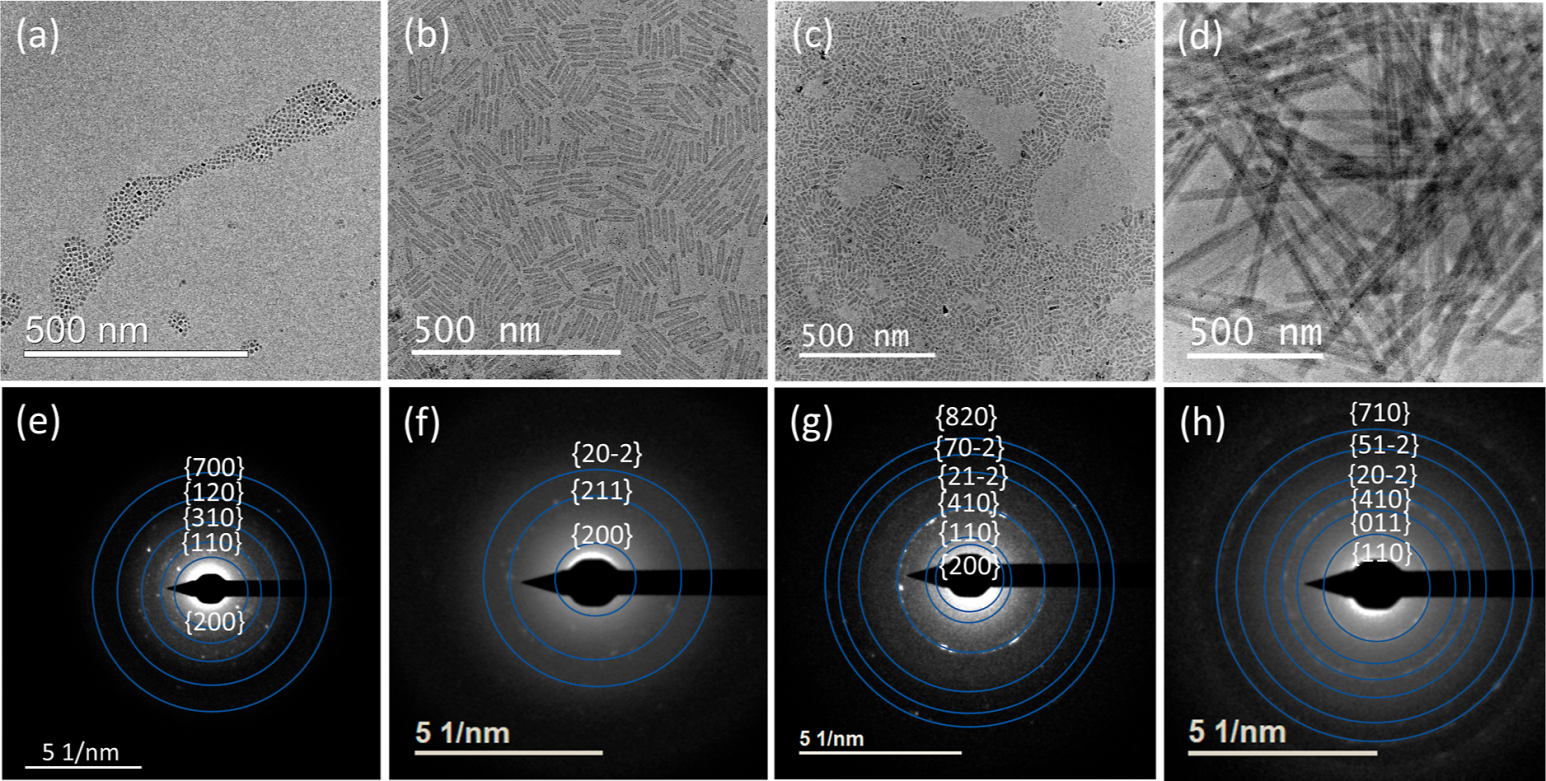
TEM micrographs and SAED of colloidal heterostructures using the deprotonated precursors with variations of reaction and growth temperature. (a) At room temperature in acetone, (b,c) at 150 °C for 5 min with applied heating in the complexation step, and (d) at 180 °C for 5 min with applied heating in the complexation step. (e), (f), (g), and (h) are SAED of (a), (b), (c), and (d), respectively, matching the reported heterostructure diffractogram.

More symmetric nanoparticles can be achieved by following the second synthesis route using the deprotonated precursor and growing them at room temperature. This is shown in Figure 4a and e, presenting a TEM micrograph and SAED of the nanoparticles, respectively (a size distribution is shown in Figure S2b). Unlike the previous synthesis (Figure 2a), small and uniform nanocubes are formed at room temperature. We hypothesize that this difference is based on the presence of water molecules, which enter the synthesis by adding HBr (in an aqueous solution). This stage, missing in the previous synthesis, aims to achieve the heterostructure with the deprotonated precursor. Zhang et al.^33^ showed that small amounts of water added to the synthesis could replace some of the DMSO in the [PbBr_4_DMSO_2_]^2–^ complex. The small water molecules replace the bulky DMSO and thus reduce the steric hindrance, increasing the nucleation rate by reducing Δ*G* for nucleation, thus leading to smaller nanoparticles.^33^ This hypothesis aligns with classical nucleation theory and is schematically shown in Figure S2f. However, although the particles produced by the deprotonated route are cubic and dispersed, the synthesis yield is low compared with the protonated route, where the growth occurs at higher temperatures. In passing, we note that adding residual water affects the preferred synthesis byproduct (from oleylamine to AMTP monolayers). This is explained in more detail in the Supporting Information.

To improve the synthesis yield, heating was applied in the complexation step (mixing the precursors in the DMSO). The results are presented in Figures 4, S3 and S4. In Figure S3, different mixing times after the injection of the DMSO solution are shown, exhibiting an increase in the particle size, as shown in the size distributions in Figure S5. The same change was also made to the deprotonated route at high temperatures (150 and 180 °C), as seen in Figures 4 and S4. Figures S3d–f, 4f–h, and 4Sb show ring pattern SAED of all particles. In all of these cases, we achieve a higher quantity of particles with decreased size, probably due to the heating of the complexation step. This increases the amount of both complexes or the relative amount of the complex that is least favored at room temperature, enabling more heterostructured nuclei to be created. In both routes, the same trend is achieved. When synthesizing at room temperature (in acetone), we get cubic-shaped particles that differ in their size, probably due to the presence of water (Figures 2a and 4a). When synthesizing at 150 or 180 °C (in ODE), we get nanoplates/nanorods (Figures S3c and 4b–d). Following other studies reporting changes in particle size and shape due to concentration and ratio of reagents,^34−36^ precursor type,^37^ coordinating solvent type,^34^ and acidity (acid–base interactions),^38^ our hypnosis is that the change seen here is due to changes in the complex’s ratio and concentration. The summary of all of the synthesis parameters changed is presented in Table S4.

**Figure 5:**
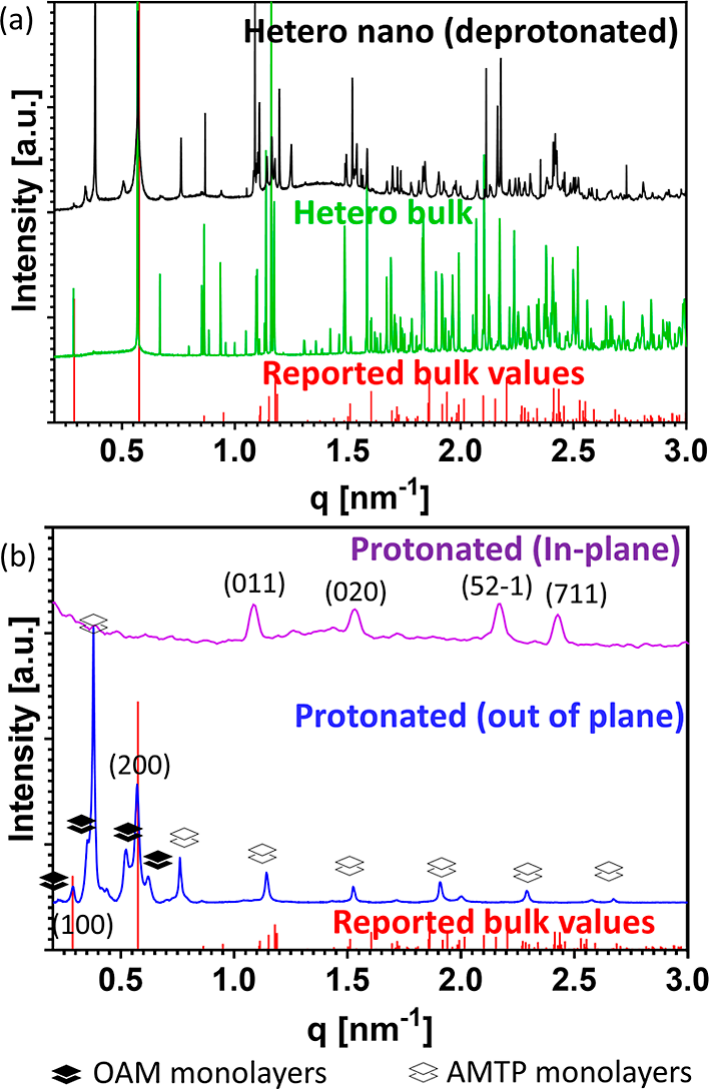
Structural analysis of colloidal heterostructures. Synchrotron diffractograms of (a) bulk (green) and heterostructure nanoparticles using deprotonated precursors (black) matching reported values (red),^28^ with a competing phase of AMTP monolayers. (b) XRD measurements of the colloidal heterostructure using protonated precursors measured in the out of plane mode (blue) and in-plane mode (purple), matching reported values marked with red lines.^28^ The competing phases of the AMTP/OAM monolayers are marked. Here, we favor the AMTP monolayer using a hydrated precursor.

The structure validation of the products of both syntheses is presented in Figure 5, showing diffractograms of the colloidal heterostructure nanoparticles compared to bulk and matching to the (PbBr_2_)_2_(AMTP)_2_PbBr_4_ heterostructure-reported values.^28^ This is demonstrated for the deprotonated and protonated precursors (Figure 5a,b, respectively). The main difference between panels a and b stems from the experimental setups. Figure 5a data show measurements taken on a synchrotron high-resolution powder diffraction beamline, while Figure 5b is derived by a lab-based diffractometer. Additional peaks in these samples are attributed to AMTP monolayers, which are competing byproducts in the synthesis. These data are validated using DFT simulations and Rietveld refinement to better distinguish this structure from the heterostructure (characterization of the monolayers is detailed in the Supporting Information). In Figure 5b, we perform two types of measurements to better characterize the products and byproducts of our colloidal synthesis. We conducted an in-plane diffraction scan in addition to the normal out of plane one. In the in-plane diffraction mode, the X-ray source and detector move parallel to the sample plane, probing 2θχ while keeping φ constant. This mode predominantly probes in-plane *d*-spacings, in contrast to the out of plane mode. Combining both techniques, we can assign the stacked AMTP and OAM monolayer byproduct diffraction peaks, marked with white/black squares, respectively. The assignment of the byproduct diffraction peaks enables us to better resolve the heterostructure diffraction peaks and match them to the reported values, further validating our results.

**Figure 6:**
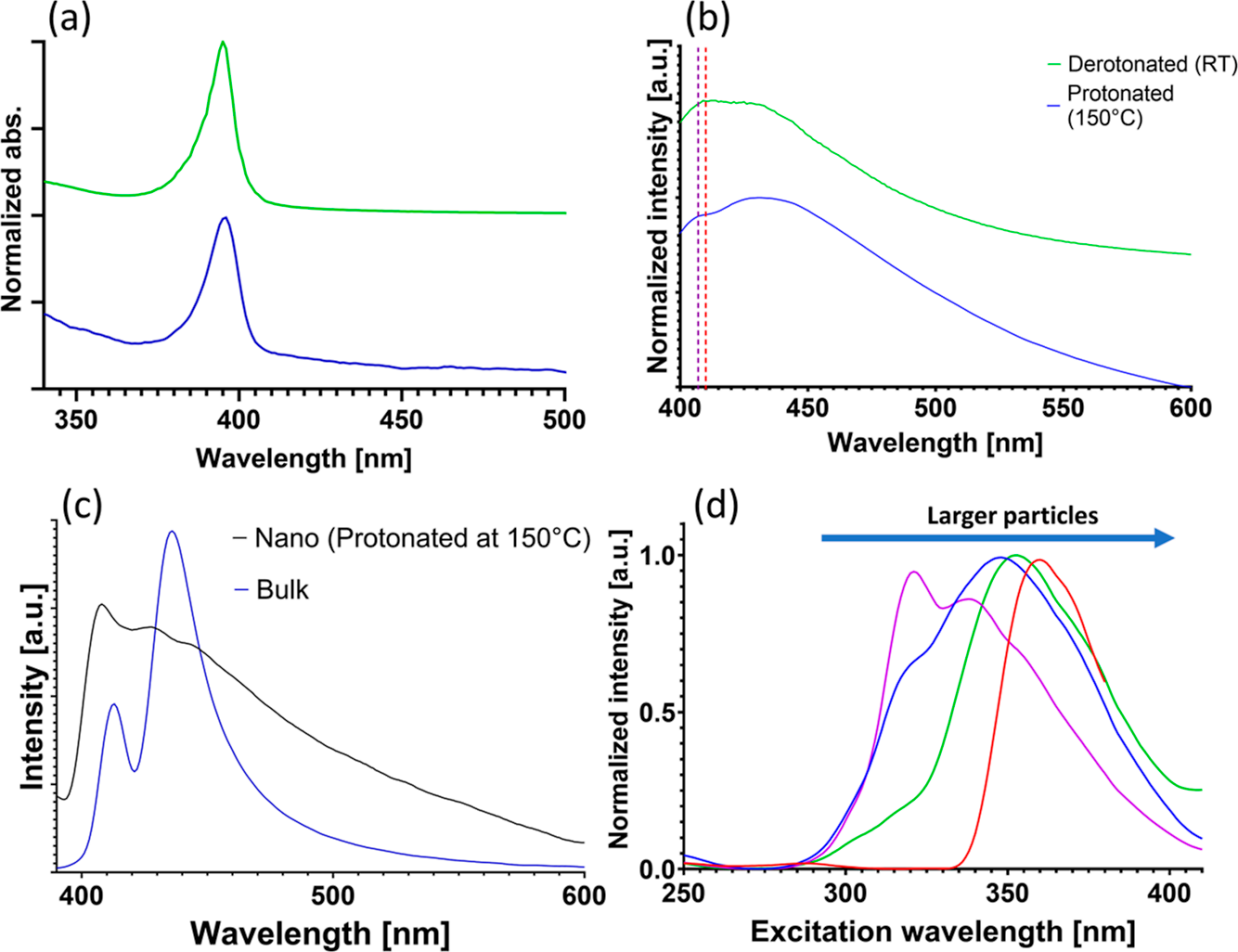
Optical characterizations of the protonated and deprotonated syntheses. (a) Absorption and (b) PL measurements of the protonated (blue) and deprotonated (green) syntheses. Graphs are shifted for clarity. The dotted lines present the central wavelength of the AMTP (red) and OAM (purple) monolayers PL. (c) PL measurements of the nano colloidal sample of the protonated route performed at 150 °C and the bulk sample at the 340 nm excitation wavelength. (d) PLE (of the 420 nm emission) of different samples showing a blue-shift as a function of particle size (purple—at 180 °C, blue—5 min at 150 °C, green—5 min at 180 °C (all using the deprotonated route and DMSO heating), and blue—protonated route at 150 °C).

In periodic heterostructures, the electrostatic potential landscape dictates the modified optoelectronic properties. UV–vis and PL spectroscopies of the colloidal heterostructure nanoparticles present a single sharp excitonic absorption peak and a broad emission peak, as shown in Figure 6a,b, respectively. This differs from the spectra of bulk heterostructure samples showing a distinct double peak (Figure S9a). Similar spectroscopic signatures were reported in the past for organic–inorganic perovskite having two emission peaks.^39−53^ The exact mechanism for this split peak is not clear and was assigned previously to photon reabsorption^39,41,51,54,55^ but also to a range of phenomena, including sub-band defect states recombination,^40,52,54−56^ spin–orbit coupling,^50,54,57,58^ and more.

Our results measure broader emissions for colloidal heterostructures compared with bulk samples. We hypothesize that these differences stem from sample homogeneity, size effects, defects, or strains (leading to broader distributions and peaks). However, there may be other explanations, such as different dielectric environments (dielectric screening affecting the exciton behavior), or surface/defect states that are more pronounced due to the high surface-to-volume ratio in colloidal samples.^40,45,46,54,59−62^

Although the controlled colloidal heterostructures present unique attributes, similar spectral features remain between the bulk and colloidal samples. For example, the PL–PLE maps in Figures S10 and S11 show similar excitation wavelengths in the colloidal samples compared to the bulk (Figure S12). All samples show two excitation ranges (above 300 nm; see the Supporting Information for a lower excitation range). With a 340 nm central wavelength range (as seen in Figure 6c) and a slight blue-shift in the second range of about 10 nm (360 nm in the nanoparticle sample compared to 370 nm in the bulk sample). These similarities probably originate from the periodic quantum well structure of the material, creating quantum confinement common to both nanocrystalline and bulk samples.

The PL characteristics of the synthesized nanocrystals, particularly those produced with a heated complexation step, are shown in Figure S9b. This figure compares the PL spectra of various syntheses with those of a protonated method where complexation was performed at room temperature without heating. It is observed in Figure S9b that samples with larger particle sizes, corresponding to the size distributions shown in Figures S2e and S5, exhibit similar PL spectra. Conversely, two samples containing smaller particles display a noticeable red-shift in the central wavelength of their spectra.

Additionally, upon examination of the PL–PLE maps in Figures S10 and S11 and the PL measurements in Figure 6d, there is a discernible blue-shift in the excitation ranges for these syntheses when compared to both the bulk material and nanoparticles synthesized without the application of heat during complexation, which are of larger size. The blue-shift in smaller particles is likely attributed to quantum confinement effects occurring along the *y*- or *z*-axis. Notably, quantum confinement is inherently present in the *x*-axis, even in the bulk material, due to its layered structure.

In addition, the PLQY of the heterostructure nanoparticles was measured to be 1.46–1.93%, similar to the bulk values of 1.12–2.83%,^29^ and correlated with other organic perovskite colloidal nanoparticles of the same scale of PLQY.^63,64^ A cautionary note on these results is in line due to the high optical density of the measured samples, which can hamper PLQY data. Reducing the concentration of future samples is an ongoing challenge due to the instability of the nanoparticles once they are diluted.

Despite the small differences between the nanoparticles and bulk, we will mention that the advantages of colloidal samples are their accessible extended emission features and the ability to control their size and shape more easily than the bulk, for example, by tuning synthesis parameters. This should be shown in more depth in future studies of the colloidal heterostructure.

To separate the optical characterization of the heterostructure nanoparticles and monolayer byproducts, samples containing each monolayer type were synthesized and measured, as seen in Figure S14 and marked in Figures 6b and S9b. The dotted lines in Figures 6b and S9b represent the central PL wavelength of the byproduct monolayers, showing overlap with part of the heterostructure PL. To solve this issue, PL–PLE maps of each monolayer sample were measured (Figure S14), showing their sharp PL peak and different optical features compared to the heterostructure samples, proving that the maps in S10 and S11 contain optical features that can be attributed to the heterostructure nanoparticles and not the monolayer byproducts (see the Supporting Information for further comparison and discussion).

## 4 Conclusions

In conclusion, a colloidal synthesis for a periodic heterostructure nanoparticle is demonstrated. We show that selecting the precursors’ protonation state is critical for the purity of the products. These results, together with the synthetic temperature dependence, match the classical nucleation theory common in a typical colloidal synthesis. The ability to control the nanocrystal size together with a narrow size distribution depends on fine-tuning the precursor concentrations to keep the synthesis in the size-focusing regime. We believe such a step is critical to further utilizing the advantages of the optical properties of both colloidal nanocrystals and bulk samples. The vision is that this first report will drive more research into studies of perovskite periodic heterostructures.

## Supporting Information Available

Monolayers, heterostructure size distributions, additional XRD, and optical measurements (PDF)

## Abbreviations

AMTP: 4-(ammoniomethyl)-tetrahydropyran
DFT: density functional theory
DMSO: dimethyl sulfoxide
HBr: hydrobromic acid
HR-PXRD: high-resolution powder X-ray diffraction
HRSTEM: high-resolution scanning transmission electron microscope
OA: oleic acid
OAM: oleyl amine
OAM-Br: oleyl ammonium bromide
ODE: octadecene
PL: photoluminescence
PLE: photoluminescence excitation
PLQY: photoluminescence quantum yield
RT: room temperature
SAED: selected area electron diffraction
TEM: transmission electron microscope
UV–vis: ultraviolet–visible range
XRD: X-ray diffraction

## References

[ref1] Liu X.; Hersam M. C. Interface Characterization and Control of 2D Materials and Heterostructures. Adv. Mater. 2018, 30 (39), 1801586 10.1002/adma.201801586. 30039558

[ref2] Yang M.-M.; Luo Z.-D.; Mi Z.; Zhao J.; E S. P.; Alexe M. Piezoelectric and Pyroelectric Effects Induced by Interface Polar Symmetry. Nature 2020, 584 (7821), 377–381. 10.1038/s41586-020-2602-4. 32814890

[ref3] Li J.; Ma Y.; Li Y.; Li S. S.; An B.; Li J.; Cheng J.; Gong W.; Zhang Y. Interface Influence on the Photoelectric Performance of Transition Metal Dichalcogenide Lateral Heterojunctions. ACS Omega 2022, 7 (43), 39187–39196. 10.1021/acsomega.2c05151. 36340091 PMC9631909

[ref4] Jin K. J.; Lu H. B.; Zhao K.; Ge C.; He M.; Yang G. Z. Novel Multifunctional Properties Induced by Interface Effects in Perovskite Oxide Heterostructures. Adv. Mater. 2009, 21 (45), 4636–4640. 10.1002/adma.200901046.

[ref5] Shi E.; Gao Y.; Finkenauer B. P.; Akriti A.; Coffey A. H.; Dou L. Two-Dimensional Halide Perovskite Nanomaterials and Heterostructures. Chem. Soc. Rev. 2018, 47 (16), 6046–6072. 10.1039/C7CS00886D. 29564440

[ref6] Liu Y. H.; Wang G.; Wang R. J.; Zhao D. Q.; Pan M. X.; Wang W. H. Super Plastic Bulk Metallic Glasses at Room Temperature. Science 2007, 315 (5817), 1385–1388. 10.1126/science.1136726. 17347434

[ref7] Fang C.; Wang H.; Li D. Recent Progress in Two-Dimensional Ruddlesden-Popper Perovskite Based Heterostructures. 2D Mater. 2021, 8 (2), 022006 10.1088/2053-1583/abdbca.

[ref8] Kavokin A.; Lagoudakis P. Exciton-Polariton Condensates: Exciton-Mediated Superconductivity. Nat. Mater. 2016, 15 (6), 599–600. 10.1038/nmat4646. 27217189

[ref9] Ma Y.; Guo W.; Fan Q.; Xu H.; Tang L.; Liu Y.; Li W.; Liu X.; Luo J.; Sun Z. Non-Artificial Layered Heterostructure as Inch-Size Single Crystal for Shortwave Polarized-Light Array Detector. advance functional materials 2022, 33 (3), 2210235 10.1002/adfm.202210235.

[ref10] Shih M. C.; Li S. S.; Hsieh C. H.; Wang Y. C.; Yang H. D.; Chiu Y. P.; Chang C. S.; Chen C. W. Spatially Resolved Imaging on Photocarrier Generations and Band Alignments at Perovskite/PbI2 Heterointerfaces of Perovskite Solar Cells by Light-Modulated Scanning Tunneling Microscopy. Nano Lett. 2017, 17 (2), 1154–1160. 10.1021/acs.nanolett.6b04803. 28094957

[ref11] Roman B. J.; Otto J.; Galik C.; Downing R.; Sheldon M. Au Exchange or Au Deposition: Adv. Funct. Mater.Dual Reaction Pathways in Au-CsPbBr3 Heterostructure Nanoparticles. Nano Lett. 2017, 17 (9), 5561–5566. 10.1021/acs.nanolett.7b02355. 28759245

[ref12] Zheng Z.; Zhuge F.; Wang Y.; Zhang J.; Gan L.; Zhou X.; Li H.; Zhai T. Decorating Perovskite Quantum Dots in TiO2 Nanotubes Array for Broadband Response Photodetector. Adv. Funct. Mater. 2017, 27, 1703115 10.1002/adfm.201703115.

[ref13] Etgar L.; Gao P.; Xue Z.; Peng Q.; Chandiran A. K.; Liu B.; Nazeeruddin M. K.; Grätzel M. Mesoscopic CH 3NH 3PbI 3/TiO 2 Heterojunction Solar Cells. J. Am. Chem. Soc. 2012, 134 (42), 17396–17399. 10.1021/ja307789s. 23043296

[ref14] Jiang G.; Guhrenz C.; Kirch A.; Sonntag L.; Bauer C.; Fan X.; Wang J.; Reineke S.; Gaponik N.; Eychmüller A. Highly Luminescent and Water-Resistant CsPbBr3-CsPb2Br5 Perovskite Nanocrystals Coordinated with Partially Hydrolyzed Poly(Methyl Methacrylate) and Polyethylenimine. ACS Nano 2019, 13 (9), 10386–10396. 10.1021/acsnano.9b04179. 31430122

[ref15] Chen W.; Hao J.; Hu W.; Zang Z.; Tang X.; Fang L.; Niu T.; Zhou M. Enhanced Stability and Tunable Photoluminescence in Perovskite CsPbX3/ZnS Quantum Dot Heterostructure. Small 2017, 13, 1604085 10.1002/smll.201604085. 28407459

[ref16] Li X.; Hoffman J. M.; Kanatzidis M. G. The 2D Halide Perovskite Rulebook: How the Spacer Influences Everything from the Structure to Optoelectronic Device Efficiency. Chem. Rev. 2021, 121 (4), 2230–2291. 10.1021/acs.chemrev.0c01006. 33476131

[ref17] Zhang X.; Lu M.; Zhang Y.; Wu H.; Shen X.; Zhang W.; Zheng W.; Colvin V. L.; Yu W. W. PbS Capped CsPbI3 Nanocrystals for Efficient and Stable Light-Emitting Devices Using p- i- n Structures. ACS Cent. Sci. 2018, 4 (10), 1352–1359. 10.1021/acscentsci.8b00386. 30410973 PMC6202640

[ref18] Dirin D. N.; Dreyfuss S.; Bodnarchuk M. I.; Nedelcu G.; Papagiorgis P.; Itskos G.; Kovalenko M. V. Lead Halide Perovskites and Other Metal Halide Complexes as Inorganic Capping Ligands for Colloidal Nanocrystals. J. Am. Chem. Soc. 2014, 136 (18), 6550–6553. 10.1021/ja5006288. 24746226 PMC4524702

[ref19] Sytnyk M.; Yakunin S.; Schöfberger W.; Lechner R. T.; Burian M.; Ludescher L.; Killilea N. A.; Yousefiamin A.; Kriegner D.; Stangl J.; Groiss H.; Heiss W. Quasi-Epitaxial Metal-Halide Perovskite Ligand Shells on PbS Nanocrystals. ACS Nano 2017, 11 (2), 1246–1256. 10.1021/acsnano.6b04721. 28135069

[ref20] Dutta S. K.; Bera S.; Pradhan N. Why Is Making Epitaxially Grown All Inorganic Perovskite-Chalcogenide Nanocrystal Heterostructures Challenging? Some Facts and Some Strategies. Chem. Mater. 2021, 33 (11), 3868–3877. 10.1021/acs.chemmater.1c01000.

[ref21] Imran M.; Peng L.; Pianetti A.; Pinchetti V.; Ramade J.; Zito J.; Di Stasio F.; Buha J.; Toso S.; Song J.; Infante I.; Bals S.; Brovelli S.; Manna L. Halide Perovskite-Lead Chalcohalide Nanocrystal Heterostructures. J. Am. Chem. Soc. 2021, 143 (3), 1435–1446. 10.1021/jacs.0c10916. 33440926 PMC7844828

[ref22] Xuan T.; Lou S.; Huang J.; Cao L.; Yang X.; Li H.; Wang J. Monodisperse and Brightly Luminescent CsPbBr3/Cs4PbBr6 Perovskite Composite Nanocrystals. Nanoscale 2018, 10 (21), 9840–9844. 10.1039/C8NR01266K. 29785438

[ref23] Ren L.; Wang M.; Wang S.; Zhang Z.; Jin K. Enhanced Photoresponsive Properties of Perovskite Films on Metal Oxide LaAlO3 Substrates. J. Phys. Chem. C 2018, 122 (19), 10495–10500. 10.1021/acs.jpcc.8b03309.

[ref24] Lin X.; Jumabekov A. N.; Lal N. N.; Pascoe A. R.; Gómez D. E.; Duffy N. W.; Chesman A. S. R.; Sears K.; Fournier M.; Zhang Y.; Bao Q.; Cheng Y. B.; Spiccia L.; Bach U. Dipole-Field-Assisted Charge Extraction in Metal-Perovskite-Metal Back-Contact Solar Cells. Nat. Commun. 2017, 8 (1), 613 10.1038/s41467-017-00588-3. 28931833 PMC5606993

[ref25] Pan D.; Fu Y.; Spitha N.; Zhao Y.; Roy C. R.; Morrow D. J.; Kohler D. D.; Wright J. C.; Jin S. Deterministic Fabrication of Arbitrary Vertical Heterostructures of Two-Dimensional Ruddlesden-Popper Halide Perovskites. Nat. Nanotechnol. 2021, 16 (2), 159–165. 10.1038/s41565-020-00802-2. 33257896

[ref26] Shi E.; Yuan B.; Shiring S. B.; Gao Y.; Akriti; Guo Y.; Su C.; Lai M.; Yang P.; Kong J.; Savoie B. M.; Yu Y.; Dou L. Two-Dimensional Halide Perovskite Lateral Epitaxial Heterostructures. Nature 2020, 580 (7805), 614–620. 10.1038/s41586-020-2219-7. 32350477

[ref27] Chen J.; Morrow D. J.; Fu Y.; Zheng W.; Zhao Y.; Dang L.; Stolt M. J.; Kohler D. D.; Wang X.; Czech K. J.; Hautzinger M. P.; Shen S.; Guo L.; Pan A.; Wright J. C.; Jin S. Single-Crystal Thin Films of Cesium Lead Bromide Perovskite Epitaxially Grown on Metal Oxide Perovskite (SrTiO3). J. Am. Chem. Soc. 2017, 139 (38), 13525–13532. 10.1021/jacs.7b07506. 28872870

[ref28] Aubrey M. L.; Saldivar Valdes A.; Filip M. R.; Connor B. A.; Lindquist K. P.; Neaton J. B.; Karunadasa H. I. Directed Assembly of Layered Perovskite Heterostructures as Single Crystals. Nature 2021, 597 (7876), 355–359. 10.1038/s41586-021-03810-x. 34526708

[ref29] Zu H.-Y.; Han X.-B.; Fan C.-C.; Liang B.-D.; Zhang W. [(4AMTP)PbBr _2_] _2_ PbBr _4_: A Nontypical Cation-Coordinated Perovskite Showing Deep-Blue Emissions and Blue-Light Photoelectric Response. Inorg. Chem. 2022, 61 (44), 17738–17745. 10.1021/acs.inorgchem.2c02900. 36284508

[ref30] Kresse G.; Hafner J. Ab. *Ab initio*molecular dynamics for liquid metals. Phys. Rev. B 1993, 47, 558–561. 10.1103/PhysRevB.47.558. 10004490

[ref31] Kresse G.; Hafner J. Ab Initio Molecular-Dynamics Simulation of the Liquid-Metal-Amorphous-Semiconductor Transition in Germanium. Phys. Rev. B 1994, 49, 14251–14269. 10.1103/physrevb.49.14251. 10010505

[ref32] Grimme S.; Antony J.; Ehrlich S.; Krieg H. A Consistent and Accurate Ab Initio Parametrization of Density Functional Dispersion Correction (DFT-D) for the 94 Elements H-Pu. J. Chem. Phys. 2010, 132 (15), 154104 10.1063/1.3382344. 20423165

[ref33] Zhang K.; Wang Z.; Wang G.; Wang J.; Li Y.; Qian W.; Zheng S.; Xiao S.; Yang S. A Prenucleation Strategy for Ambient Fabrication of Perovskite Solar Cells with High Device Performance Uniformity. Nat. Commun. 2020, 11 (1), 1006 10.1038/s41467-020-14715-0. 32081847 PMC7035260

[ref34] Jancik Prochazkova A.; Scharber M. C.; Yumusak C.; Jančík J.; Másilko J.; Brüggemann O.; Weiter M.; Sariciftci N. S.; Krajcovic J.; Salinas Y.; Kovalenko A. Synthesis Conditions Influencing Formation of MAPbBr3 Perovskite Nanoparticles Prepared by the Ligand-Assisted Precipitation Method. Sci. Rep. 2020, 10, 15720 10.1038/s41598-020-72826-6. 32973262 PMC7518261

[ref35] Grisorio R.; Conelli D.; Giannelli R.; Fanizza E.; Striccoli M.; Altamura D.; Giannini C.; Allegretta I.; Terzano R.; Suranna G. P. A New Route for the Shape Differentiation of Cesium Lead Bromide Perovskite Nanocrystals with Near-Unity Photoluminescence Quantum Yield. Nanoscale 2020, 12 (32), 17053–17063. 10.1039/D0NR04246C. 32785320

[ref36] Dutta A.; Dutta S. K.; Das Adhikari S.; Pradhan N. Tuning the Size of CsPbBr3 Nanocrystals: All at One Constant Temperature. ACS Energy Lett. 2018, 3 (2), 329–334. 10.1021/acsenergylett.7b01226.

[ref37] Chakrabarty A.; Satija S.; Gangwar U.; Sapra S. Precursor-Mediated Synthesis of Shape-Controlled Colloidal CsPbBr3 Perovskite Nanocrystals and Their Nanofiber-Directed Self-Assembly. Chem. Mater. 2020, 32 (2), 721–733. 10.1021/acs.chemmater.9b03700.

[ref38] Almeida G.; Goldoni L.; Akkerman Q.; Dang Z.; Khan A. H.; Marras S.; Moreels I.; Manna L. Role of Acid-Base Equilibria in the Size, Shape, and Phase Control of Cesium Lead Bromide Nanocrystals. ACS Nano 2018, 12 (2), 1704–1711. 10.1021/acsnano.7b08357. 29381326 PMC5830690

[ref39] Yamada T.; Yamada Y.; Nakaike Y.; Wakamiya A.; Kanemitsu Y. Photon Emission and Reabsorption Processes in CH3NH3PbBr3 Single Crystals Revealed by Time-Resolved Two-Photon-Excitation Photoluminescence Microscopy. Phys. Rev. Appl. 2017, 7 (1), 014001 10.1103/PhysRevApplied.7.014001.

[ref40] Birkhold S. T.; Zimmermann E.; Kollek T.; Wurmbrand D.; Polarz S.; Schmidt-Mende L. Impact of Crystal Surface on Photoexcited States in Organic-Inorganic Perovskites. Adv. Funct. Mater. 2017, 27, 1604995 10.1002/adfm.201604995.

[ref41] Schötz K.; Askar A. M.; Peng W.; Seeberger D.; Gujar T. P.; Thelakkat M.; Köhler A.; Huettner S.; Bakr O. M.; Shankar K.; Panzer F. Double Peak Emission in Lead Halide Perovskites by Self-Absorption. J. Mater. Chem. C 2020, 8 (7), 2289–2300. 10.1039/C9TC06251C.

[ref42] Steele J. A.; Puech P.; Monserrat B.; Wu B.; Yang R. X.; Kirchartz T.; Yuan H.; Fleury G.; Giovanni D.; Fron E.; Keshavarz M.; Debroye E.; Zhou G.; Sum T. C.; Walsh A.; Hofkens J.; Roeffaers M. B. J. Role of Electron-Phonon Coupling in the Thermal Evolution of Bulk Rashba-Like Spin-Split Lead Halide Perovskites Exhibiting Dual-Band Photoluminescence. ACS Energy Lett. 2019, 4 (9), 2205–2212. 10.1021/acsenergylett.9b01427.

[ref43] Niesner D.; Schuster O.; Wilhelm M.; Levchuk I.; Osvet A.; Shrestha S.; Batentschuk M.; Brabec C.; Fauster T. Temperature-Dependent Optical Spectra of Single-Crystal (CH3NH3)PbBr3 Cleaved in Ultrahigh Vacuum. Phys. Rev. B 2017, 95 (7), 075207 10.1103/PhysRevB.95.075207.

[ref44] Chi X.; Leng K.; Wu B.; Shi D.; Choy Y.; Chen Z.; Chen Z.; Yu X.; Yang P.; Xu Q. H.; Sum T. C.; Rusydi A.; Loh K. P. Elucidating Surface and Bulk Emission in 3D Hybrid Organic-Inorganic Lead Bromide Perovskites. Adv. Opt. Mater. 2018, 6, 1800470 10.1002/adom.201800470.

[ref45] Murali B.; Yengel E.; Yang C.; Peng W.; Alarousu E.; Bakr O. M.; Mohammed O. F. The Surface of Hybrid Perovskite Crystals: A Boon or Bane. ACS Energy Lett. 2017, 2 (4), 846–856. 10.1021/acsenergylett.6b00680.

[ref46] Sheikh T.; Shinde A.; Mahamuni S.; Nag A. Possible Dual Bandgap in (C4H9NH3)2PbI4 2D Layered Perovskite: Single-Crystal and Exfoliated Few-Layer. ACS Energy Lett. 2018, 3 (12), 2940–2946. 10.1021/acsenergylett.8b01799.

[ref47] Sheikh T.; Nawale V.; Pathoor N.; Phadnis C.; Chowdhury A.; Nag A. Molecular Intercalation and Electronic Two Dimensionality in Layered Hybrid Perovskites. Angew. Chem., Int. Ed. 2020, 59 (28), 11653–11659. 10.1002/anie.202003509. 32243656

[ref48] Nawale V. V.; Sheikh T.; Nag A. Dual Excitonic Emission in Hybrid 2D Layered Tin Iodide Perovskites. J. Phys. Chem. C 2020, 124 (38), 21129–21136. 10.1021/acs.jpcc.0c05301.

[ref49] Dhanabalan B.; Castelli A.; Palei M.; Spirito D.; Manna L.; Krahne R.; Arciniegas M. Simple Fabrication of Layered Halide Perovskite Platelets and Enhanced Photoluminescence from Mechanically Exfoliated Flakes. Nanoscale 2019, 11 (17), 8334–8342. 10.1039/C9NR00638A. 30984951

[ref50] Niesner D.; Wilhelm M.; Levchuk I.; Osvet A.; Shrestha S.; Batentschuk M.; Brabec C.; Fauster T. Giant Rashba Splitting in CH3NH3PbBr3 Organic-Inorganic Perovskite. Phys. Rev. Lett. 2016, 117 (12), 126401 10.1103/PhysRevLett.117.126401. 27689285

[ref51] Yamada T.; Yamada Y.; Kanemitsu Y. Photon Recycling in Perovskite CH3NH3PbX3 (X = I, Br, Cl) Bulk Single Crystals and Polycrystalline Films. J. Lumin. 2020, 220, 116987 10.1016/j.jlumin.2019.116987.

[ref52] Fu J.; Jamaludin N. F.; Wu B.; Li M.; Solanki A.; Ng Y. F.; Mhaisalkar S.; Huan C. H. A.; Sum T. C. Localized Traps Limited Recombination in Lead Bromide Perovskites. Adv. Energy Mater. 2019, 9 (12), 1803119 10.1002/aenm.201803119.

[ref53] Dar M. I.; Jacopin G.; Meloni S.; Mattoni A.; Arora N.; Boziki A.; Zakeeruddin S. M.; Rothlisberger U.; Grätzel M. Origin of Unusual Bandgap Shift and Dual Emission in Organic-Inorganic Lead Halide Perovskites. Sci. Adv. 2016, 2, 1601156 10.1126/sciadv.1601156. PMC5091363 27819049

[ref54] Droseros N.; Tsokkou D.; Banerji N. Photophysics of Methylammonium Lead Tribromide Perovskite: Free Carriers, Excitons, and Sub-Bandgap States. Adv. Energy Mater. 2020, 10 (13), 1903258 10.1002/aenm.201903258.

[ref55] Lyu D.; Miao Y.; Li B.; Xiao Z.; Wu X.; Hu X.; Jiang X. F.; Xu Q. H. Dual Blue Emission in Ruddlesden-Popper Lead-Bromide Perovskites Induced by Photon Recycling. J. Phys. Chem. C 2021, 125 (33), 18308–18316. 10.1021/acs.jpcc.1c04891.

[ref56] Shi J.; Zhang H.; Li Y.; Jasieniak J. J.; Li Y.; Wu H.; Luo Y.; Li D.; Meng Q. Identification of High-Temperature Exciton States and Their Phase-Dependent Trapping Behaviour in Lead Halide Perovskites. Energy Environ. Sci. 2018, 11 (6), 1460–1469. 10.1039/C7EE03543H.

[ref57] Steele J. A.; Puech P.; Monserrat B.; Wu B.; Yang R. X.; Kirchartz T.; Yuan H.; Fleury G.; Giovanni D.; Fron E.; Keshavarz M.; Debroye E.; Zhou G.; Sum T. C.; Walsh A.; Hofkens J.; Roeffaers M. B. J. Role of Electron-Phonon Coupling in the Thermal Evolution of Bulk Rashba-Like Spin-Split Lead Halide Perovskites Exhibiting Dual-Band Photoluminescence. ACS Energy Lett. 2019, 4 (9), 2205–2212. 10.1021/acsenergylett.9b01427.

[ref58] Hu S.; Gao H.; Qi Y.; Tao Y.; Li Y.; Reimers J. R.; Bokdam M.; Franchini C.; Di Sante D.; Stroppa A.; Ren W. Dipole Order in Halide Perovskites: Polarization and Rashba Band Splittings. J. Phys. Chem. C 2017, 121 (41), 23045–23054. 10.1021/acs.jpcc.7b05929.

[ref59] Shi E.; Deng S.; Yuan B.; Gao Y.; Akriti; Yuan L.; Davis C. S.; Zemlyanov D.; Yu Y.; Huang L.; Dou L. Extrinsic and Dynamic Edge States of Two-Dimensional Lead Halide Perovskites. ACS Nano 2019, 13 (2), 1635–1644. 10.1021/acsnano.8b07631. 30812095

[ref60] Tran M. D.; Kim J. H.; Lee Y. H. Tailoring Photoluminescence of Monolayer Transition Metal Dichalcogenides. Curr. Appl. Phys. 2016, 16 (9), 1159–1174. 10.1016/j.cap.2016.03.023.

[ref61] Ghanta U.; Ray M.; Biswas S.; Sardar S.; Maji T. K.; Pal S. K.; Bandyopadhyay N. R.; Liu B.; Hossain S. M. Effect of Phonon Confinement on Photoluminescence from Colloidal Silicon Nanostructures. J. Lumin. 2018, 201, 338–344. 10.1016/j.jlumin.2018.04.052.

[ref62] Zhou J.; Li L.; Gui Z.; Buddhudu S.; Zhou Y. Photoluminescence of CdSe Nanocrystallites Embedded in BaTiO3Matrix. Appl. Phys. Lett. 2000, 76 (12), 1540–1542. 10.1063/1.126089.

[ref63] Thirumal K.; Chong W. K.; Xie W.; Ganguly R.; Muduli S. K.; Sherburne M.; Asta M.; Mhaisalkar S.; Sum T. C.; Soo H. S.; Mathews N. Morphology-Independent Stable White-Light Emission from Self-Assembled Two-Dimensional Perovskites Driven by Strong Exciton-Phonon Coupling to the Organic Framework. Chem. Mater. 2017, 29 (9), 3947–3953. 10.1021/acs.chemmater.7b00073.

[ref64] Weidman M. C.; Seitz M.; Stranks S. D.; Tisdale W. A. Highly Tunable Colloidal Perovskite Nanoplatelets through Variable Cation, Metal, and Halide Composition. ACS Nano 2016, 10 (8), 7830–7839. 10.1021/acsnano.6b03496. 27471862

