# Supplementary material for: Colloidal Synthesis of (PbBr2)2(AMTP)2PbBr4 a Periodic Perovskite “Heterostructured” Nanocrystal
Source: Cryst Growth Des. 2024 Mar 26;24(8):3237–45. doi: 10.1021/acs.cgd.3c01472 (PMC11036359; doi:10.1021/acs.cgd.3c01472)
Supplement: Supplementary file 1 — cg3c01472_si_001.pdf [file cg3c01472_si_001.pdf]

Supporting information

Colloidal synthesis of $(PbBr_2)_2(AMTP)_2PbBr_4$ A periodic perovskite "heterostructured" nanocrystal

Emma H. Massasa[1], Lotte T. J. Kortstee[3], Rachel Lifer[1], Saar Shaek[1], Boaz Pokroy[1], Ivano E. Castelli[3], Yehonadav Bekenstein[1,2]

[1] Department of Materials Science and Engineering, Technion – Israel Institute of Technology, 32000 Haifa, Israel.

[2] The Solid-state institute, Technion – Israel Institute of Technology, 32000 Haifa, Israel.

[3] Department of Energy Conversion and Storage (DTU Energy), Technical University of Denmark, Anker Engelunds Vej 411, DK-2800 Kongens Lyngby, Denmark.

AMTP precursor

The heterostructure was synthesized in two different synthetic routes, using the protonated and deprotonated precursor. The differences between the two routes are reflected in the AMTP molecule. In the deprotonated precursor route, the AMTP molecule (Fig. S1a) is added to the heterostructure synthesis with the addition of HBr. While in the protonated precursor route, the AMTP molecule is first protonated (Fig. S1b) and then added to the heterostructure synthesis.

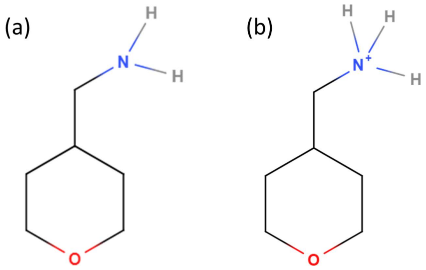

Figure S1. (a) Chemical structure of the deprotonated AMTP molecule. (b) Chemical structure of the protonated AMTP molecule

Heterostructure synthesis

Size distributions of all syntheses were done, and the length and width of the different particles were measured as presented in Figs. S2 and S5, showing a clear trend of the decrease in size. When comparing Fig. S2a and b, we can see a decrease in particle size as a result of the reaction's hydration. Fig S2a presents the size distribution of the colloidal heterostructure nanoparticles synthesized using the protonated precursor. Fig. S2b presents the size distribution of the colloidal heterostructure nanoparticles synthesized using the deprotonated precursor. One of the main differences between the two synthetic routes is the addition of HBr in aqueous solution. Adding water to the solution affects the complexes in the synthesis and can increase the nucleation rate, creating smaller nanoparticles. As discussed by Zhang et al., the water molecules can replace the DMSO in the $[PbBr_4DMSO_2]^{-2}$ complex and thus decrease its steric hindrance, increasing the nucleation rate and reducing the particle size[1].

An additional synthetic parameter that was changed in the heterostructure synthesis is the temperature of the synthesis using the protonated precursor. Fig. S2 a, c-e presents the size distributions as the temperature increases, showing a decrease in the size of the nanoparticles due to the decrease in the critical size of the nuclei. The influence of the two effects, hydration, and temperature, on the critical radii is schematically demonstrated on a classic nucleation theory curve in Fig. S2f. Both effects decrease the energy required for nucleation, decreasing the critical radius of nucleation, thus forming more nuclei and producing smaller nanoparticles as a result, enabling us to control the synthesis and the final product.

Moreover, we can see that increasing the amount of the protonated precursor changes the shape of the nanoparticles to nanorods or nanoplates (as seen in Fig. 2). This is also shown in the size distribution of the width compared to the length of the nanoparticles in Fig. S2d and e, as we see a small shift in the width compared to the length (compared to Fig. S2a-c). The reason for the change in particle shape is still unknown.

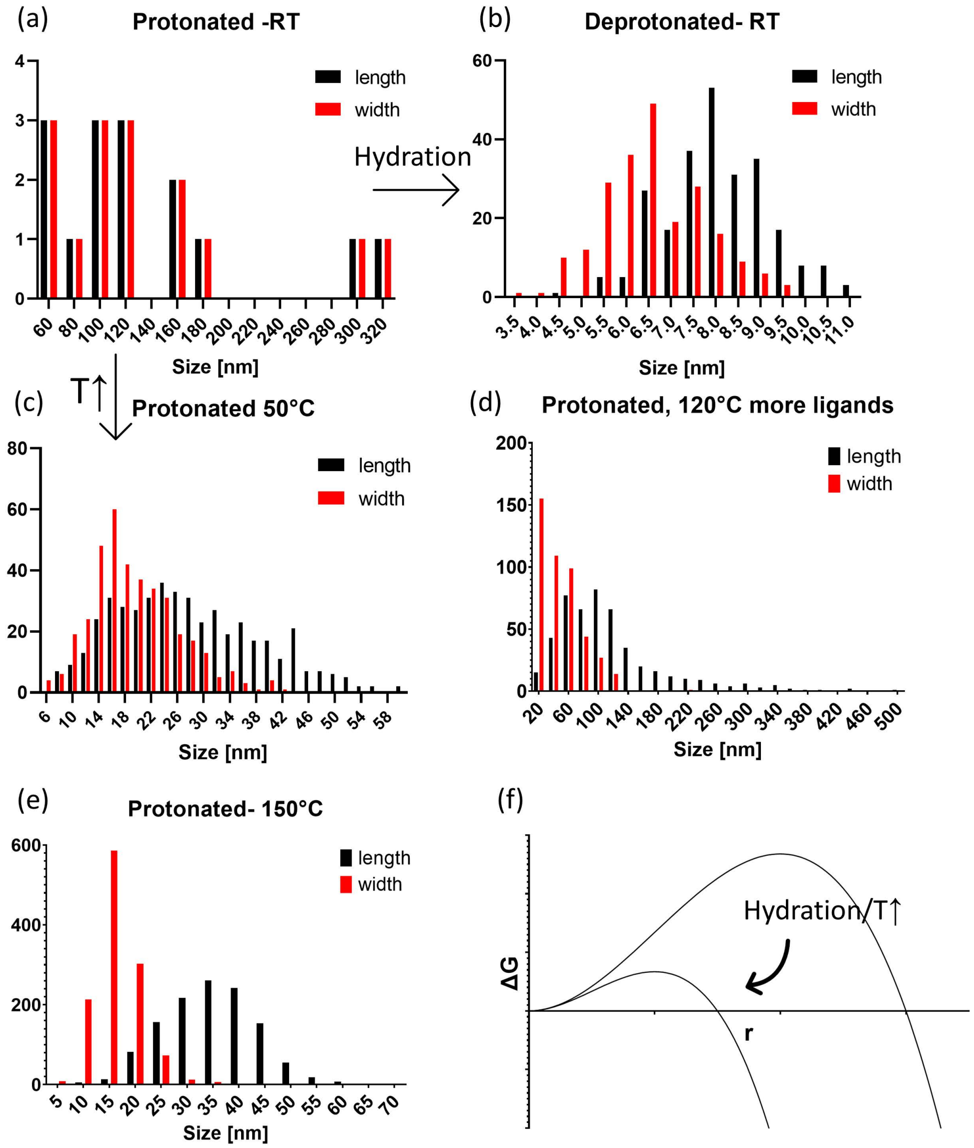

Figure S2. (a), (b), (c), (d), and (e) are size distributions of colloidal heterostructures using protonated precursors at room temperature, deprotonated precursors at room temperature, protonated precursors at 50˚C, protonated precursors at 120˚C with more ligands (and more AMTP) and protonated precursors at 150˚C respectively. (f) is classical nucleation curve showing the change in nucleation rate and critical nuclei radius

To increase the synthesis yield, heating was applied to the complexation step (dissolving of the precursors in DMSO). This was done to increase the complex's quantity and induce the formation of both complexes needed to form the heterostructure. Fig. S3 presents the result of this change on the protonated route at 150˚C, showing an increase in particle quantity and a decrease in particle size, as seen in Fig S5. The decrease in particle size can be explained by classic nucleation theory where the increase in complexes quantity increases the number of nuclei formed, thus creating smaller particles with decreased size. In addition, we can see that as the mixing time increases, the particles grow and form rod-shaped particles. The SAED ring patterns in Fig S3d-f match the heterostructure reported values[2].

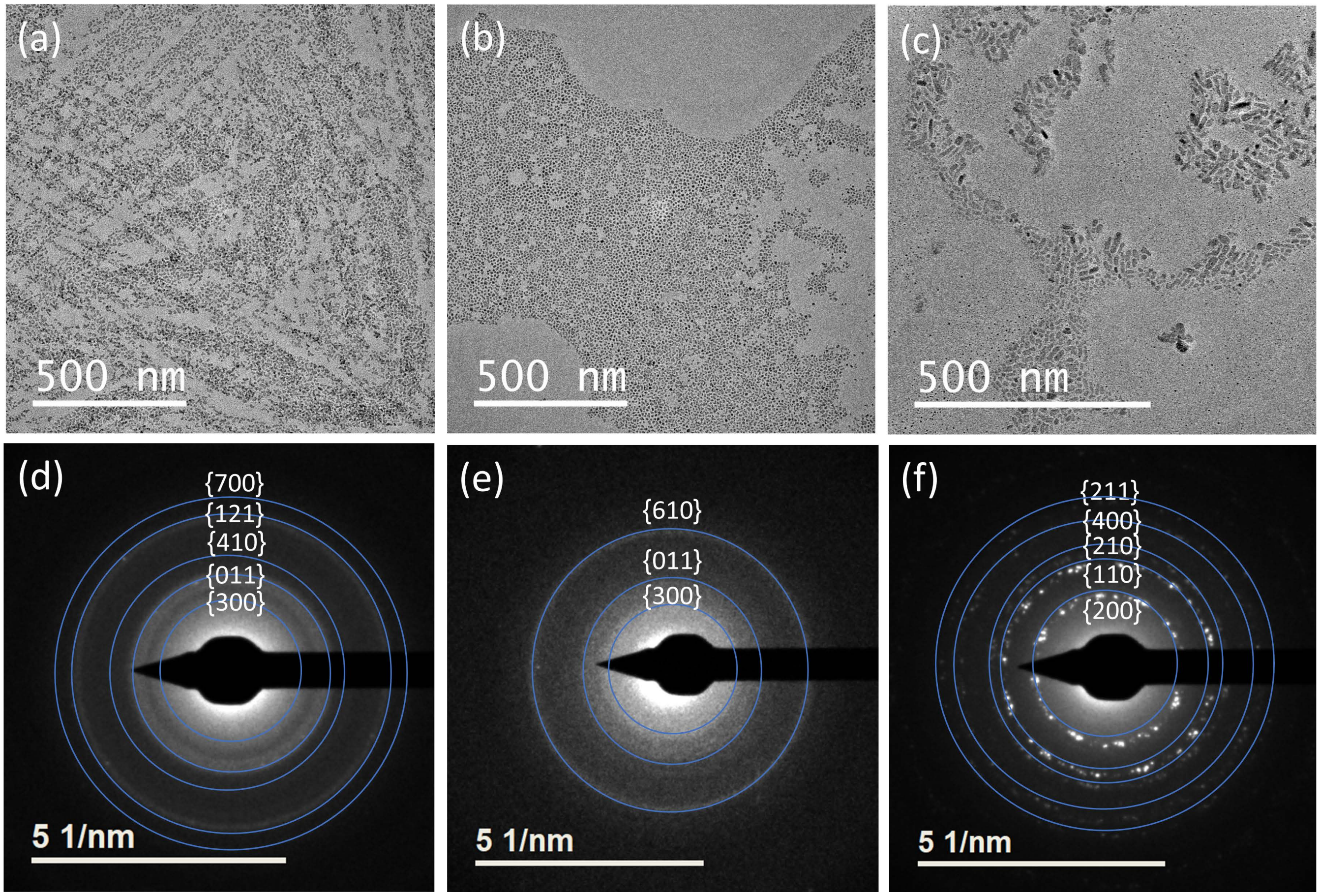

Figure S3. TEM micrographs of the synthesis using the protonated route with heating in the complexation step (DMSO) while changing the mixing time in ODE at 150C. (a) 30 seconds (b) 5 minutes (c) 15 minutes. (d), (e) and (f) are ring patterns of SAED of (a), (b), and (c), respectively

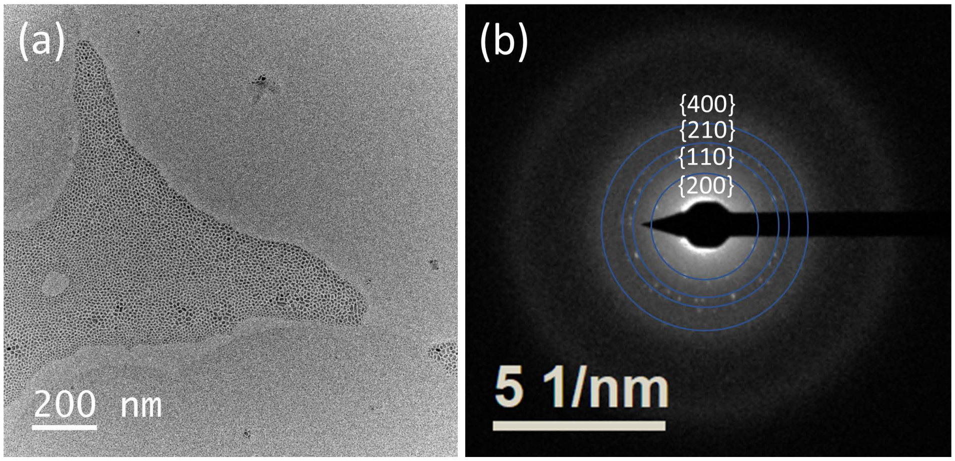

Figure S4. (a) TEM micrograph of the heterostructure using the deprotonated precursors at 180˚C with applied heating in the complexation step, and (b) is the ring pattern SAED of the particles in (a).

The same heating was also applied to the deprotonated route, with an additional change of temperature and solvent of the synthesis (done in ODE at 150˚C and 180˚C). The results in Fig. 4b and c show two populations found after 5 minutes of mixing, following the injection of the precursor solutions into ODE. When mixing the synthesis at 180˚C, we can achieve small nanoparticles (with minimal mixing at 180˚C) as seen in Fig. S4a and matching SAED (Fig. S4b), or larger particles shown in Fig. 4d when mixing for 5 minutes at 180˚C. SAED of all the nanoparticles are presented in Fig. 4e-h and S4b and match the reported values of the heterostructure[2]. Size distributions are detailed in Fig. S5.

As seen in almost all syntheses, the size distribution is not uniform, which may result from the synthesis type used to create these particles. Transitioning the synthesis to hot injection and cooling may solve this problem and should be done in the future.

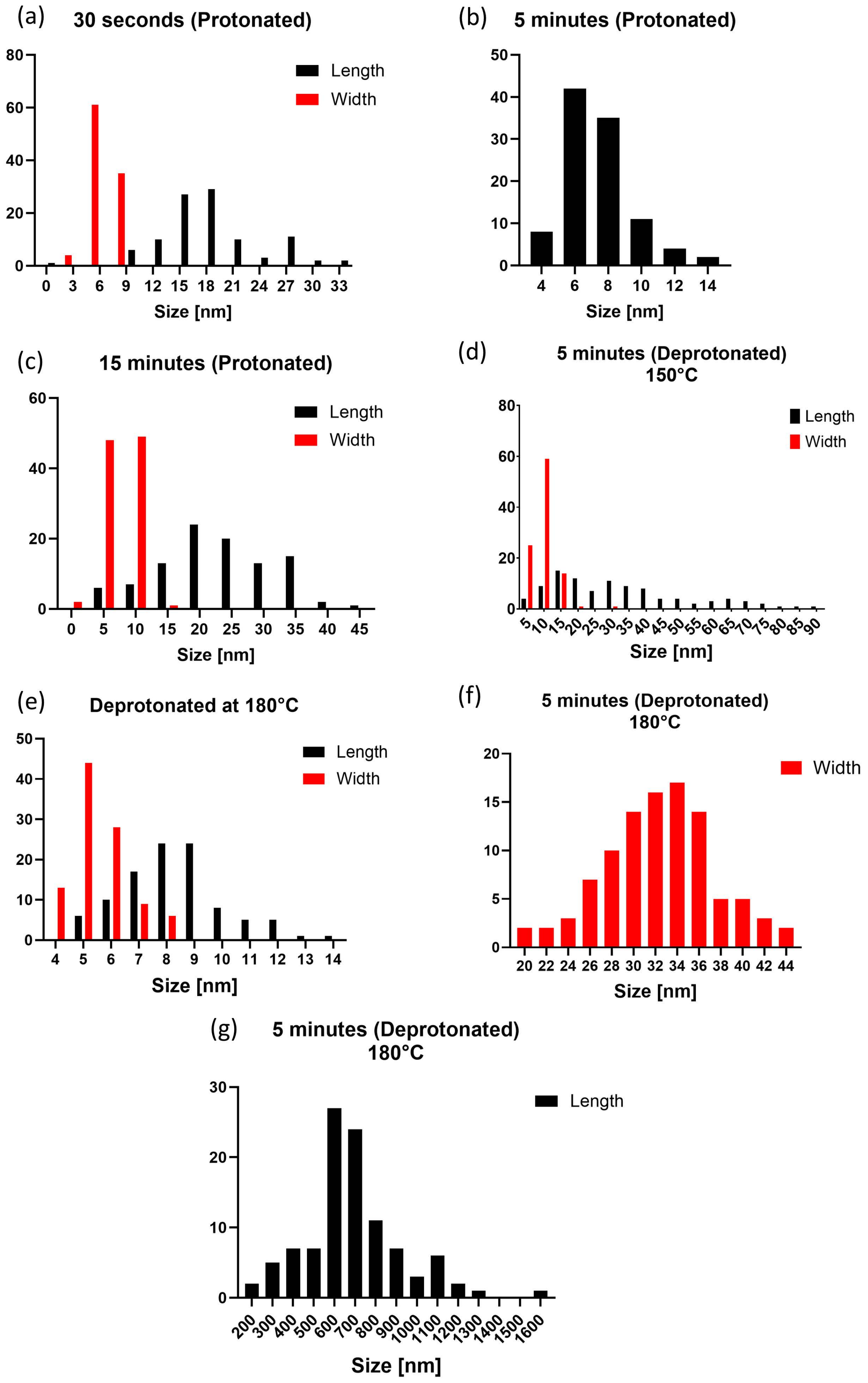
(a)
30 seconds (Protonated)
Length
Width
Size [nm]
(b)
5 minutes (Protonated)
Size [nm]
(c)
15 minutes (Protonated)
Length
Width
Size [nm]
(d)
5 minutes (Deprotonated)
150°C
Length
Width
Size [nm]
(e)
Deprotonated at 180°C
Length
Width
Size [nm]
(f)
5 minutes (Deprotonated)
180°C
Width
Size [nm]
(g)
5 minutes (Deprotonated)
180°C
Length
Size [nm]

Figure S5. (a), (b) and (c) are size distributions of colloidal heterostructures using the protonated precursors (at 150˚C), with applied heating at the complexation step, and mixing after the injection for 30 seconds, 5 minutes, and 15 minutes, respectively. (d) and (e) are size distributions of colloidal heterostructures using the deprotonated route at 150˚C and 180 ˚C, respectively, with applied heating at the complexation step, and mixing for 5 minutes after injection. (f) and (g) are the width and length size distributions of colloidal heterostructures using the deprotonated route in ODE at 180 ˚C with applied heating at the complexation step and mixing for 5 minutes after injection.

Monolayers

The colloidal heterostructure nanoparticle synthesis (in both routes) also creates by-products of monolayers. The monolayer is one layer of $PbBr_4$ octahedral that is stabilized with ligands surrounding the perovskite layer, having the ammonium head of the ligand in the A site of the perovskite. Two types of monolayers created in the synthesis are oleyl amine (OAM) monolayers and AMTP monolayers, which have the chemical formulas of OAM_2PbBr_4 and $AMTP_2PbBr_4$, respectively. The two monolayers are presented in Fig. S6a and b, and Fig. S6c shows a TEM micrograph of the monolayers stacked one on top of the other. Fig. S8a presents Synchrotron data of a powder sample of the AMTP monolayer and the colloidal heterostructure nanoparticles, showing colliding peaks of the AMTP monolayers in the heterostructure nanoparticle sample (peaks that do not match the reported values of the heterostructure), proving its existence as a by-product in the heterostructure synthesis. When the monolayers are drop-casted on a substrate, a stacking structure is formed. This creates multiple diffraction peaks with constant spacing between them in XRD measurements[3], as seen in Fig. S7 and Fig S8b. These peaks are due to the stacking structure creating a pattern of organic and inorganic layers, one on the other.

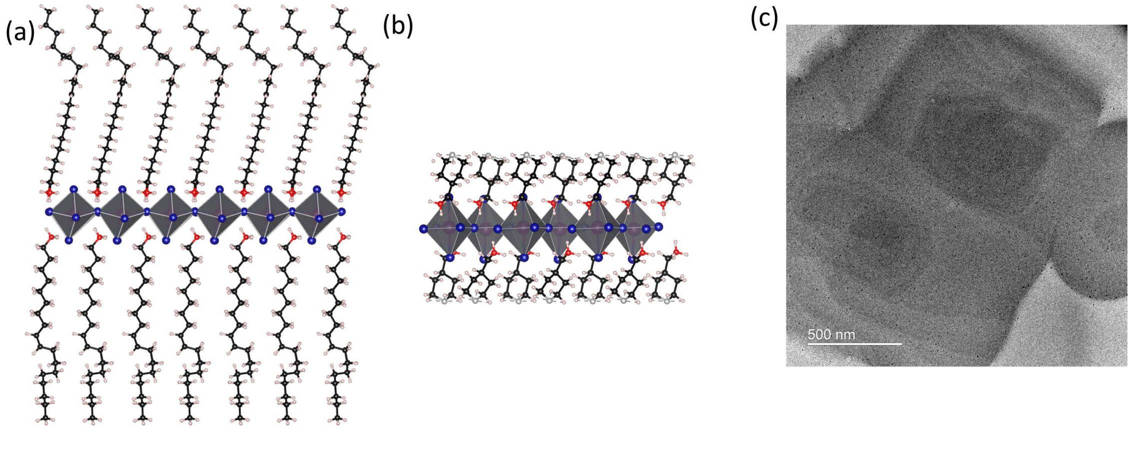

Figure S6. (a) OAM monolayers (b) AMTP monolayers and (c) TEM micrograph of the by-product monolayers

Fig. S8b also presents that the two different routes of the heterostructure synthesis have different preferred monolayer by-products. The synthesis using the deprotonated precursor prefers the AMTP monolayers, and the synthesis using the protonated precursor prefers the OAM monolayer, as shown in Fig. S8b in the green and black measurements, respectively. However, when we use the protonated precursor without drying it, meaning a wet version of the protonated precursor, the preference changes from the OAM monolayers to the AMTP monolayers. This can be explained by the same reason discussed above: the synthesis's hydration. When water is present, it can change the DMSO in the $[PbBr_4DMSO_2]^{-2}$ complex, reducing the steric hindrance of the complex and thus enabling bulkier molecules such as the AMTP (compared to the OAM) to get closer. In addition, the water molecule in the complex may also create hydrogen bonding[1], in our case, with the AMTP molecules, and so favor it to be the stabilizing ligand of the perovskite monolayers created. This effect of switching the preferred monolayer by-product by hydrating the synthesis is presented in Fig. S8b, showing the wet protonated precursor in the blue XRD measurement, with more pronounced AMTP monolayer peaks, compared to the dry protonated precursor in the black XRD measurement, with only OAM monolayer peak present.

We will mention that by measuring the constant distance between the monolayer's peaks in the XRD measurement (Fig. S8b) and by using Bragg's law, we can measure the thickness of each one of the monolayers and compare the theoretical values, as shown in Eq. 1 and 2:

$$(1)\quad d_{AMTP\ monolayers} = \frac{n\lambda}{2sin\theta} = \frac{0.154}{2\ sin\ 2.68°} = 1.6\ nm$$

$$(2)\quad d_{OAM\ monolayers} = \frac{n\lambda}{2\sin\theta} = \frac{0.154}{2\ \sin\ 1.2°} = 3.6\ nm$$

To achieve an accurate CIF file of the AMTP monolayers, a relaxed CIF was first created using the DFT relaxation workflow as described in the method section. Then, a powder sample of the AMTP monolayers was measured at the Synchrotron. By using Rietveld refinement (on the synchrotron data), we were able to refine the calculated CIF file and create a more accurate CIF of the AMTP monolayers matching the

results better. This CIF also showed a good match to results achieved with a different sample preparation (drop casting the colloidal solution on a substrate), showing a stacked structure of the monolayers, as shown in Fig. S7 and done on the Rigaku SmartLab XRD. The refinement details are shown in Table S1.

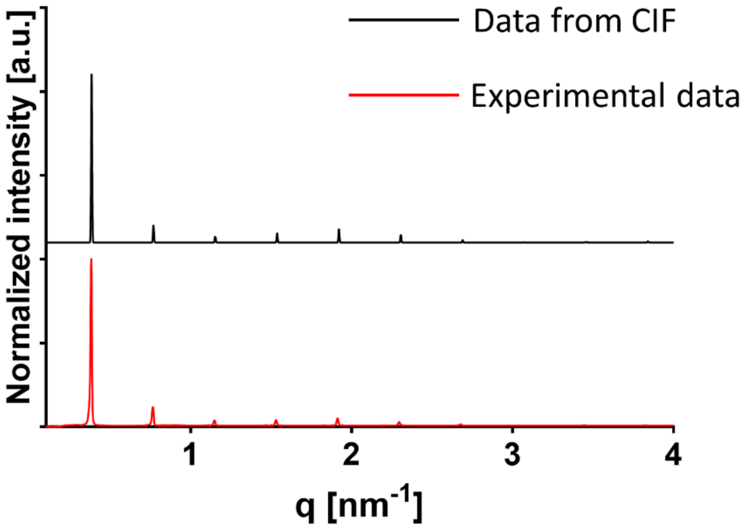

Figure S7. XRD measurement (red) and simulation (black) of a stacked structure of AMTP monolayers made by drop casting the colloidal solution on a glass substrate.

Table S1. Crystallographic data of the AMTP monolayers from the Rietveld refinement parameters

**Parameter**	
**Crystal system**	triclinic
**Space group**	P1
**a, b, c (Å)**	16.834, 8.2509, 8.2021
**α, β, γ (˚)**	89.987, 100.564, 90.22
**V [[Å^3]**	1119.89
**wR**	24.778%

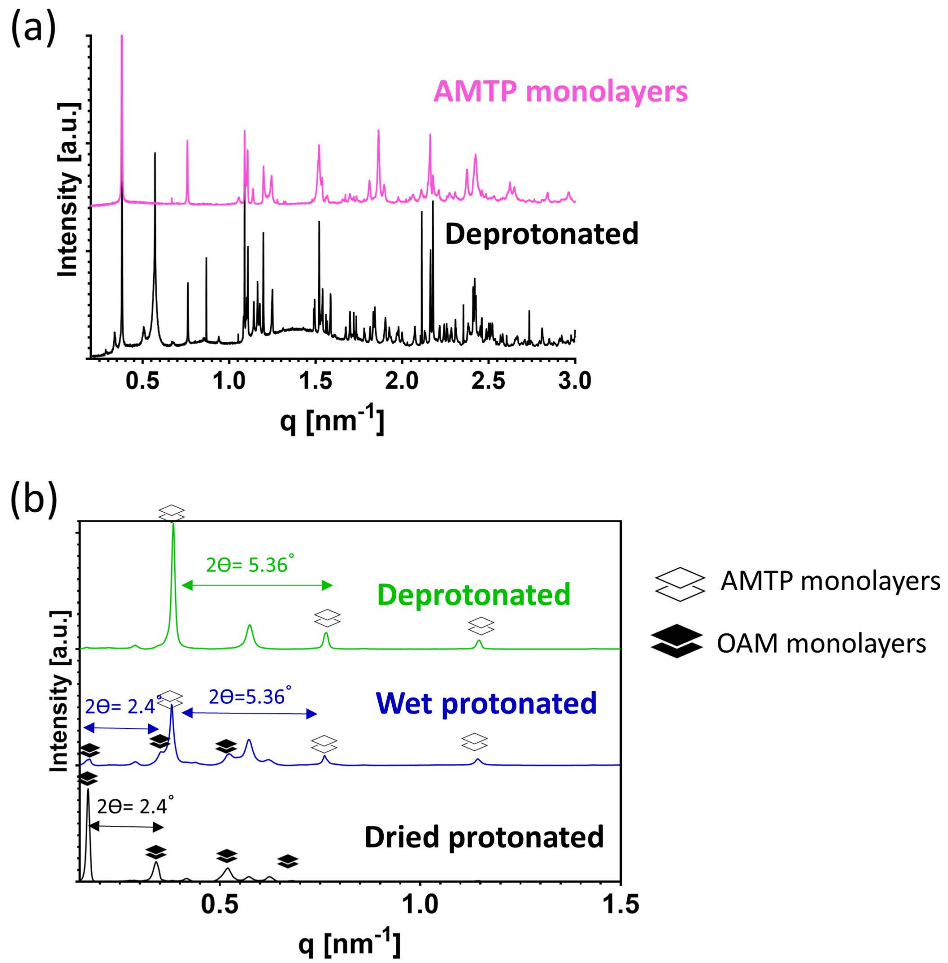

Figure S8. XRD measurements of (a) deprotonated nano heterostructure and AMTP monolayers synchrotron measurements and (b) comparison between three syntheses using dehydrated precursor (protonated) (black), hydrated precursor (protonated) (blue) and deprotonated synthesis (green).

Optical characterizations

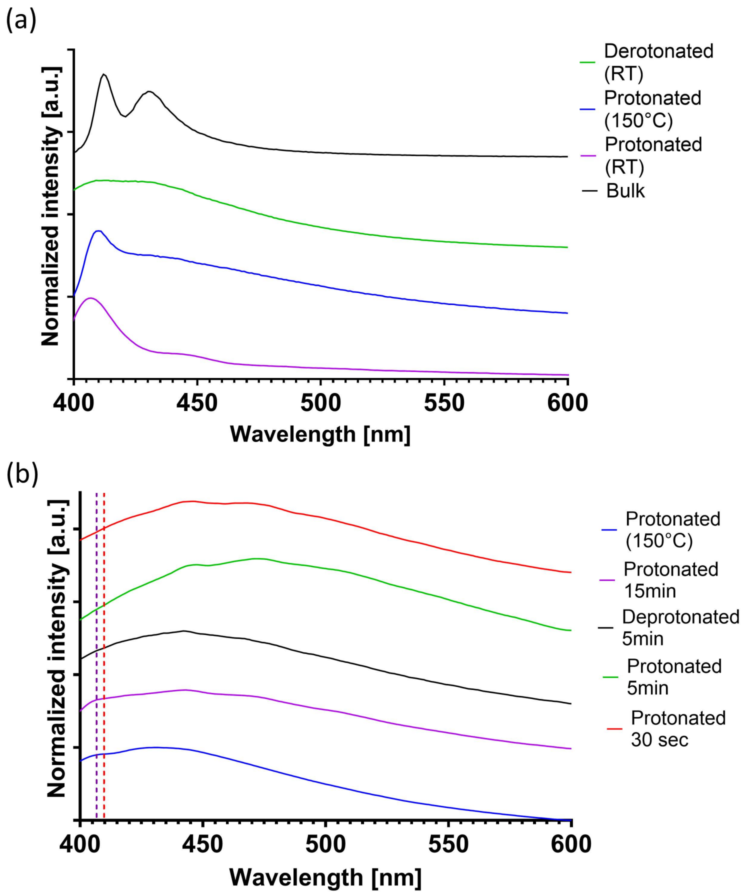

Figure S9. (a) PL measurements of the colloidal heterostructure using the deprotonated route at room temperature (green), using the protonated route at room temperature (purple), using the protonated route at 150˚C (blue), and bulk sample (black). (b) PL measurements of the colloidal heterostructure using the protonated route at 150 ˚C (blue) compared to DMSO heating syntheses of the protonated route mixed for 15 minutes (purple), 5 minutes (green), 30 seconds (red), and deprotonated route mixing for 5 minutes (black) all at 150˚.

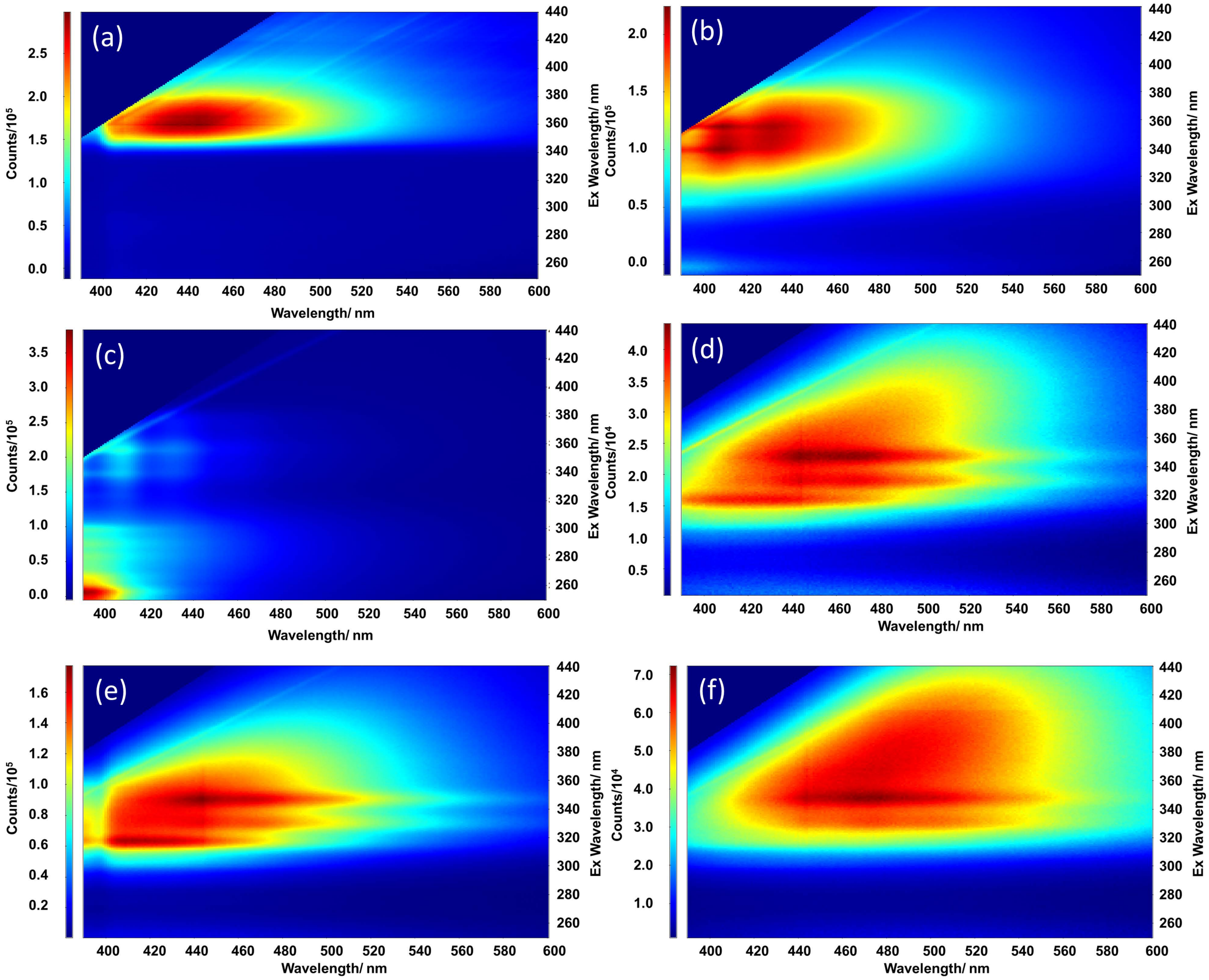

Figure S10. PL-PLE maps of colloidal heterostructure syntheses using the Protonated precursor (a) at 150˚C, (b) at 150˚C after cleaning, (c) at room temperature, (d), (e), and (f) are at 150 ˚C for 30 seconds, 5 minutes and 15 minutes, respectively, with applied heating at the complexation step.

Fig. S10-S12 presents PL-PLE maps of the different heterostructure samples showing similarities between them. The similarities between bulk (Fig. S12) and nanoparticle samples are less noticeable in Fig. S10a and S11a due to a disturbance in the background (monolayers) in Fig. S11a and an intense peak in Fig S10a. In both maps, the peaks are so intense that they ascend over the material's other peaks. The other material features are shown in Fig. S10b and c, belonging to the samples with lower product yield (Fig. S10c), or that went through an additional cleaning process, decreasing the product quantity but increasing its purity (Fig. S10b). In these cases, the other material features (excitation below 300 nm) are seen due to the more moderate intensity of the main peak. These features of excitation wavelengths at 250-290 nm

are also seen in the bulk sample (Fig. S12). We will mention that the comparison between the different maps shows that the nanoparticles present a different ratio in the intensity of the peaks, raising the intensity of the emission peak due to longer excitation wavelengths. However, this should be further studied.

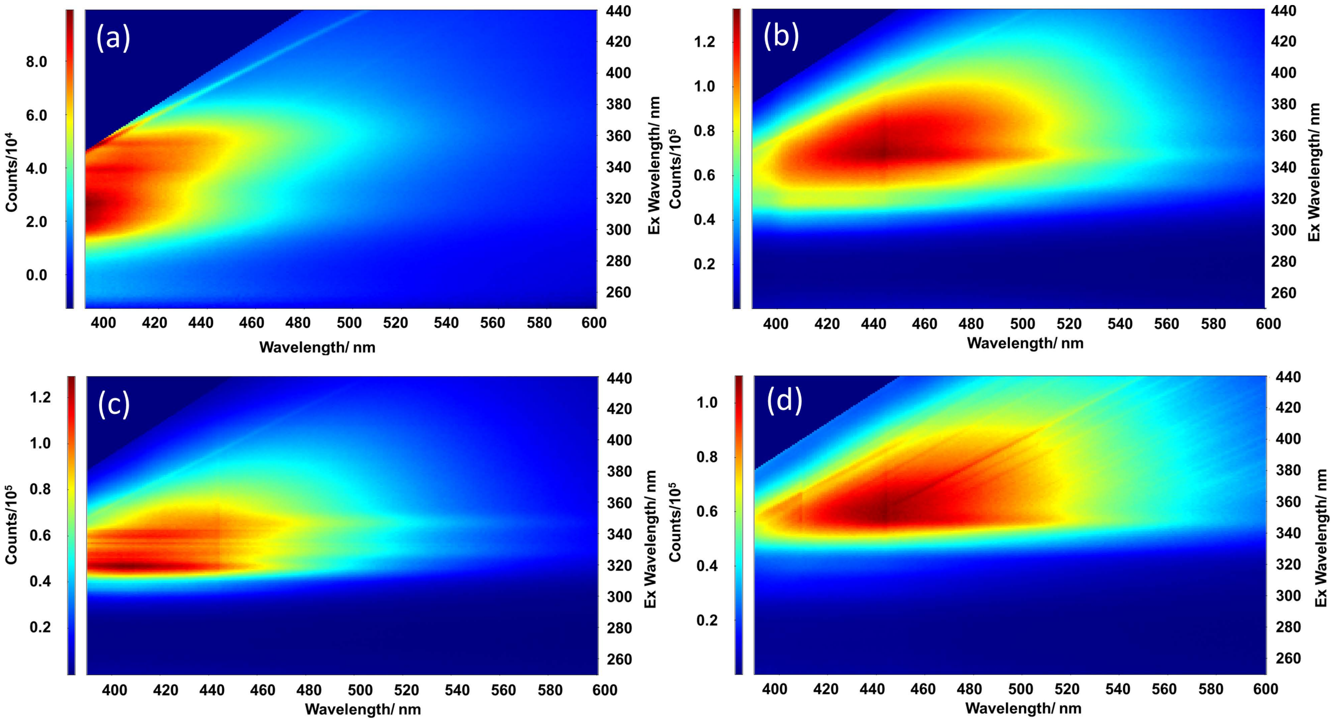

Figure S11. PL-PLE maps of colloidal heterostructure synthesis using the deprotonated precursor (a) at room temperature mixed for 30min, (b) at 150˚C mixed for 5 minutes and with applied heating to the complexation step, (c) at 180˚C and with applied heating to the complexation step (d) at 180˚C mixed for 5 minutes and with applied heating to the complexation step.

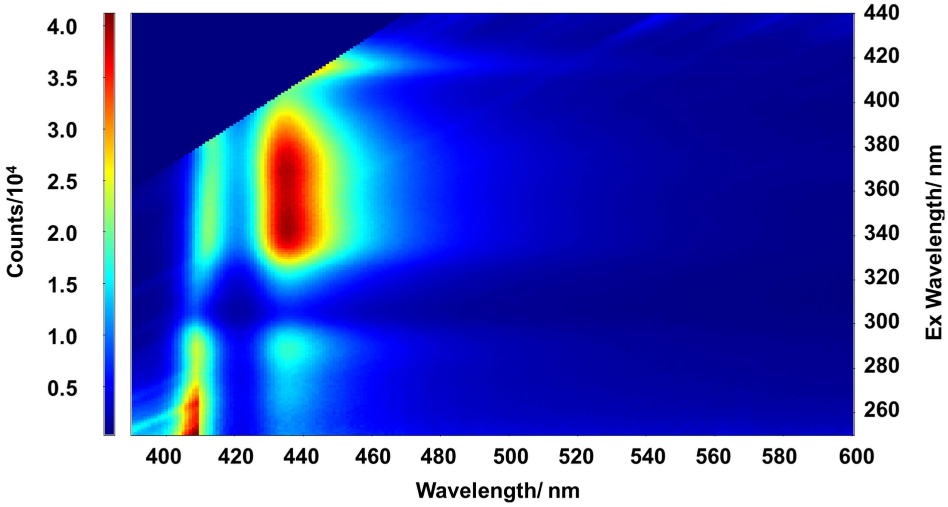

Figure S12. PL-PLE map of the bulk heterostructure

As discussed in the manuscript, the maps shown in Fig. S10-S11 of the heterostructure syntheses where the complexation step was heated show a blue shift in excitation wavelengths probably due to quantum confinement in the y or z axis, and indeed smaller size distributions are shown in theses samples (Fig. S5). In addition, we can see an additional peak (emission at around 400 nm), which we attribute to the monolayers, which is also seen in the monolayer maps in Fig S14.

Fig. S13 shows the temperature-dependent PL spectrum of the heterostructure nanoparticles compared to the OAM monolayers. We notice an additional wide red-shifted peak in both samples, possibly due to self-trapped excitons. Both samples show this feature as a result of a similar structure influencing the material's electronic structure. Furthermore, Aubrey et al.[2] calculated the electronic structure of the heterostructure bulk and showed that its electronic states are composed of only the perovskite layer.

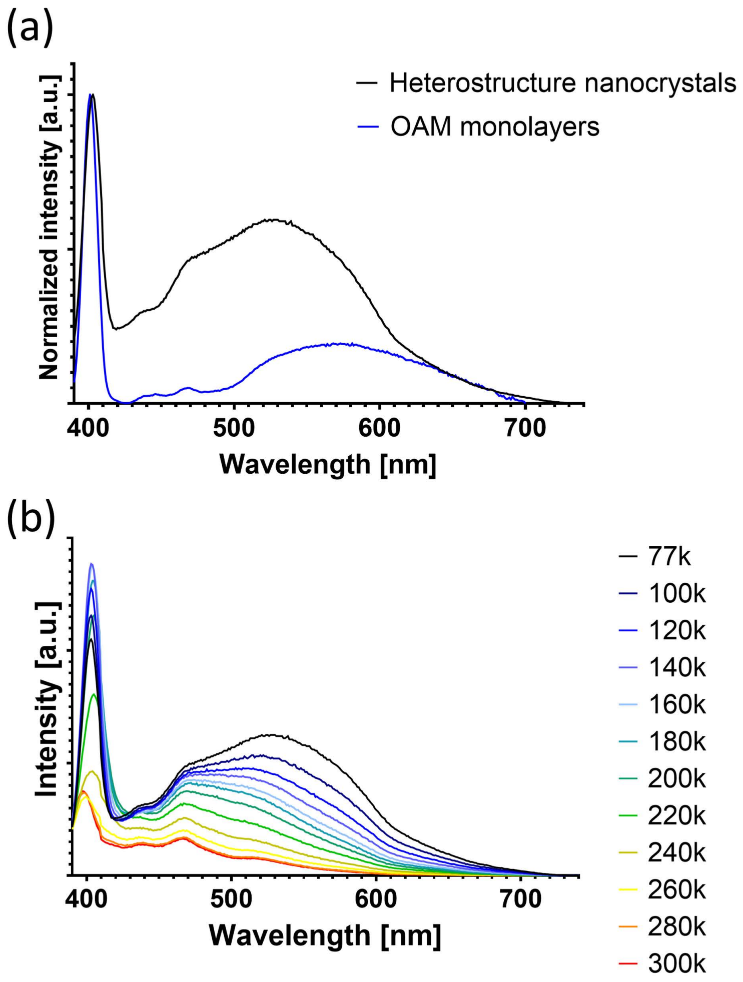

Figure S13. Temperature-dependent PL measurements of drop casted samples of (a) the heterostructure nanoparticles made via the protonated route at 150˚C (black) and OAM monolayers (blue) measured at 77K and (b) heterostructure nanoparticles synthesized via the protonated route at 150˚C.

In order to eliminate the possibility that the by-product monolayers contribute to the PL measured for the colloidal heterostructure nanoparticles, control PL measurements were done for the two samples containing only AMTP monolayers and only OAM monolayers, the data is presented in Fig. S14. The 1D PL measurement of the monolayers (Fig S14a) shows a single emission peak, at around 407 nm for the OAM monolayers and around 410 nm for the AMTP monolayers, which overlaps with the first peak present in the heterostructure 1D PL. The values of the PL peaks of each sample are summarized in Table S2. When comparing the maps of the monolayers (Fig. S14b-c) and heterostructure, differences between the samples are seen, especially in shape (range of emission wavelength) and partially in excitation wavelengths. These similarities can be explained by the existence of the perovskite layer in both monolayer and

heterostructure samples. Moreover, as mentioned before, Aubrey et al. performed DFT calculations showing the dominance of the perovskite layer on both conduction and valence band formation.[2]

Table S2. A summary of the PL peak values of the different samples

OAM monolayers	AMTP monolayers	Heterostructure bulk	Heterostructure nano
407 nm	410 nm	412 nm	400-600nm
		430 nm	

Table S3. Quantum yield of the different samples

Sample	Quantum yield
**Protonated route**	
**30 seconds**	1.7%
**5 minutes**	1.58%
**15 minutes**	1.93%
**Deprotonated route**	
**5 minutes**	1.46%

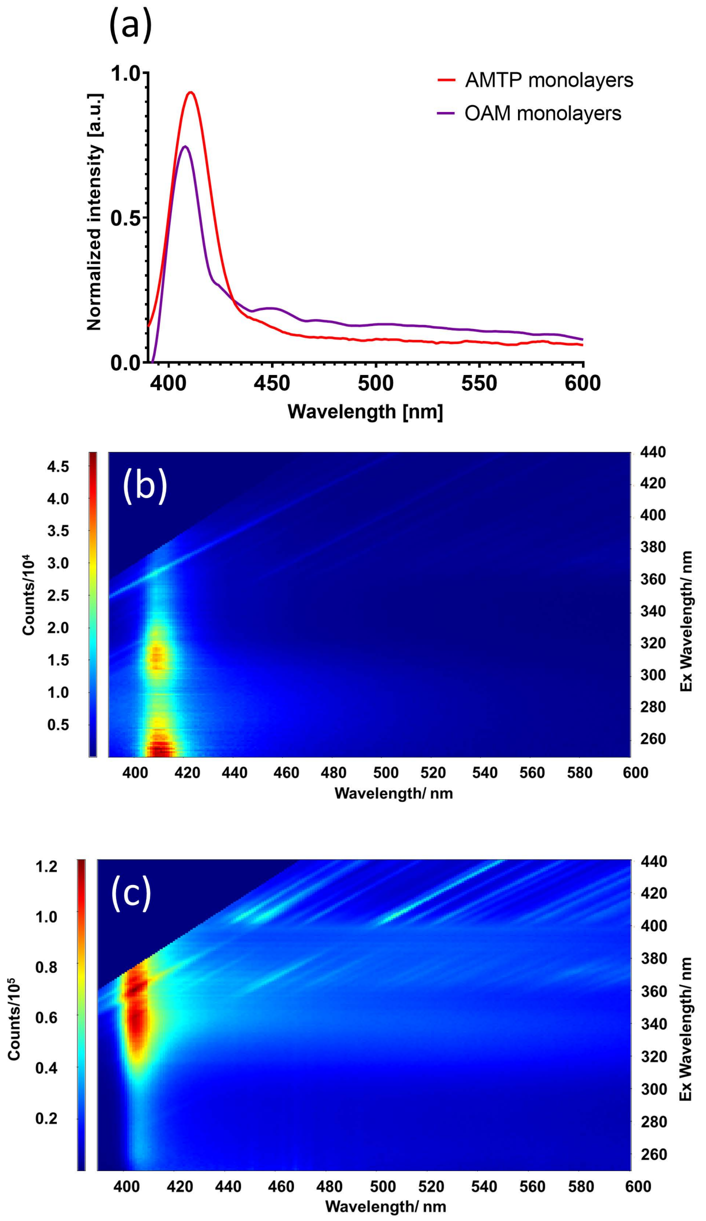

Figure S14. Optical characterization of the monolayers. (a) PL measurements of AMTP (red) and OAM (purple) monolayers. PL-PLE maps of (a) AMTP monolayers and (b) OLM monolayers showing different emission peaks and excitation wavelengths compared to the heterostructure.

Table S4. Synthesis summary

<table>
<tr><th colspan="6">Deprotonated</th></tr>
<tr><th></th><th colspan="2">0</th><th colspan="2">5 min</th><th>30 min</th></tr>
<tr><td>RT</td><td colspan="2">-</td><td colspan="2">-</td><td>7.4±1.4</td></tr>
<tr><td>150˚C</td><td colspan="2">-</td><td colspan="2">9.6±3.7 (W)
32±20 (L)</td><td>-</td></tr>
<tr><td>180˚C</td><td colspan="2">5.5±1 (W)
8.3±1.8 (L)</td><td colspan="2">32±5 (W)
688±234 (L)</td><td>-</td></tr>
<tr><th colspan="6">Protonated</th></tr>
<tr><th></th><th colspan="2">0-30sec</th><th>5 min</th><th>10 min</th><th>15 min</th></tr>
<tr><td>RT</td><td colspan="2">-</td><td>-</td><td>138±80</td><td>-</td></tr>
<tr><td>50˚C</td><td colspan="2">-</td><td>-</td><td>19±6.6 (W)
28±11 (L)</td><td>-</td></tr>
<tr><td>120˚C</td><td colspan="2">-</td><td>-</td><td>48±27 (W)
116±73 (L)</td><td>-</td></tr>
<tr><td>150˚C</td><td>16±4.2 (W)
35±8.7 (L)</td><td>6.9±1.6 (W)
17.8±5.7 (L)</td><td>7.3±2.1</td><td>-</td><td>7.7±2.1 (W)
22±9 (L)</td></tr>
</table>

* The red text indicates syntheses where the complexation step was heated

References

(1) Zhang, K.; Wang, Z.; Wang, G.; Wang, J.; Li, Y.; Qian, W.; Zheng, S.; Xiao, S.; Yang, S. A Prenucleation Strategy for Ambient Fabrication of Perovskite Solar Cells with High Device Performance Uniformity. *Nat Commun* **2020**, *11* (1), 1006.

(2) Aubrey, M. L.; Saldivar Valdes, A.; Filip, M. R.; Connor, B. A.; Lindquist, K. P.; Neaton, J. B.; Karunadasa, H. I. Directed Assembly of Layered Perovskite Heterostructures as Single Crystals. *Nature* **2021**, *597* (7876), 355–359.

(3) Toso, S.; Baranov, D.; Filippi, U.; Giannini, C.; Manna, L. Collective Diffraction Effects in Perovskite Nanocrystal Superlattices. *Acc. Chem. Res* **2023**, *56*, 67–76.
